# Mechanism of action of alkaloids in *Fritillaria*-induced autophagy and apoptosis in non-small cell lung cancer

**DOI:** 10.3892/ol.2026.15598

**Published:** 2026-04-15

**Authors:** Mengru Teng, Mengrui Zhang, Jun He, Wai Ming Tse, Kathy Wai Gaun Tse, Chengwu Liu, Bengui Ye

**Affiliations:** 1 Key Laboratory of Drug-Targeting and Drug Delivery System of The Education Ministry, Sichuan Engineering Laboratory for Plant-Sourced Drug and Sichuan Research Center for Drug Precision Industrial Technology, West China School of Pharmacy, Sichuan University, Chengdu, Sichuan 610041, P.R. China; 2 Nin Jiom Medicine Manufactory (Hong Kong) Limited, Hong Kong, SAR 999077, P.R. China; 3 Department of Thoracic Surgery, West China Hospital, Sichuan University, Chengdu, Sichuan 610041, P.R. China; 4 Medical Department, Tibet University, Lhasa, Tibet Autonomous Region 850002, P.R. China

**Keywords:** non-small cell lung cancer, alkaloids in *Fritillaria*, network pharmacology. autophagy, apoptosis

## Abstract

The medicinal plant, *Fritillaria* (commonly known as beimu), exerts notable therapeutic effects on respiratory tract and lung diseases. Its active alkaloid components have exhibited efficacy against non-small cell lung cancer (NSCLC). The present study screened the anti-NSCLC protein targets of alkaloids in *Fritillaria* and validated their functional activity *in vitro.* Network pharmacology analysis indicated that alkaloids with activity against NSCLC target >300 proteins. Verticinone and ebeiedinone inhibited the proliferation of A549 and H1299 cells. After treating H1299 and A549 cells with 15 µM verticinone, the cell survival rate dropped to 75.57±4.80% and 68.9±3.06%, respectively. After treating both cell lines with 15 µM ebeiedinone, the cell survival rates dropped to 65.75±6.44% and 67±3.20%, respectively. At 30 µM, ebeiedinone could induce 26.44% of the cells to undergo apoptosis, which collectively indicates that the two compounds have anti-proliferative and induction of apoptosis effects on NSCLC cells. Additionally, the expression of LC3-II induced by ebeiedinone (30 µM) was significantly increased in the presence of the autophagy inhibitor, chloroquine, suggesting an enhancement of the autophagic flux. In summary, the findings of the present study demonstrated the significant inhibitory effect of *Fritillaria* alkaloids on NSCLC, which was closely associated with increased apoptosis and autophagy.

## Introduction

Global cancer statistics indicate that lung cancer has the highest morbidity and mortality rates worldwide (1). Lung cancer ranks second in terms of incidence worldwide and first in terms of mortality. Every year, ~1.6 million people die from lung cancer (2). Lung cancer is classified into small cell lung cancer and non-small cell lung cancer (NSCLC) based on its pathological type, with NSCLC accounting for 85% of cases (3). Currently, chemotherapy remains the primary treatment option for lung cancer. However, the overall prognosis remains poor, characterized by a short median survival time and marked toxicities and side-effects (4). A number of natural anticancer compounds have been identified and developed for therapeutic purposes, such as paclitaxel (5), curcumin (6), phloretin (7), berberine (8) and artemisinin (9). The main aim of the present study was to identify additional novel anti-NSCLC compounds with potential for drug development.

Bulbus *Fritillaria cirrhosa* D.Don, also known as chuanbeimu, is recognized as a high-quality food and medicinal ingredient, holding the highest economic value among other beimu products, including *Fritillaria Pallidiflora* Bulbus and *Fritillaria thunbergii* Miq (10,11) Alkaloids are the primary active components of *F. cirrhosa* D.Don . Isosteroidal alkaloids exhibit significant anticancer, antitussive, expectorant, anti-inflammatory and antioxidant properties, as well as other biological properties. At present, the experimental validation of the anticancer effects of *F. cirrhosa* D.Don primarily focuses on its extracts, with a few individual alkaloids reported to exhibit anti-proliferative and anti-inflammatory activities (12–14). The material basis and mechanisms of action of *F. cirrhosa* D.Don against lung cancer warrant further investigation.

The precise roles and extent of the involvement of apoptosis and autophagy in the anticancer effects of *Fritillaria* remain unclear. Apoptosis refers specifically to the orderly self-destruction of cells, a process autonomously regulated by genes that encode proteins, such as Bax, Bcl-2, caspase-3 and PARP-1, which help maintain the stability of the internal environment following stimulation under physiological conditions (15). Autophagy is the evolutionarily conserved, lysosome-dependent degradation of cytoplasmic contents (16,17) and is associated with various human diseases and physiological processes, including cancer, neurodegeneration, microbial infections and aging (18–21). Numerous anticancer drugs simultaneously induce apoptosis and autophagy to varying degrees, presenting novel directions for drug discovery and development (22,23). Chen *et al* (24) discovered that berberine, by decreasing expressions of cyclooxygenase-2 (COX-2), MMP-2, and MMP-9 in A549 cells, induces upregulation of p21 and p53 expressions, thereby exerting an inhibitory effect on tumor development. The demethylsonodione aporphine alkaloid isolated from *Corydalis saxicola* Bunting DC. can induce apoptosis of T2 tumor cells through the ROS/p38 MAPK signaling pathway (25). The present investigated the effects of apoptosis and autophagy induced by alkaloids from *Fritillaria cirrhosa* D.Don on the viability of NSCLC cells.

Network pharmacology provides a powerful approach for elucidating the multi-component, multi-target and multi-pathway mechanisms of action of traditional Chinese medicines. The present study aimed to identify the anticancer potential of alkaloids in *Fritillaria* through network pharmacology screening and calculation, as well as perform protein-protein interaction (PPI) network analysis and enrichment to identify proteins with the most significant associations for further validation, to provide a reference for subsequent *in vitro* activity verification.

## Materials and methods

### Construction of a biological target network of alkaloids for NSCLC

First, alkaloids from the literature were compared in the Traditional Chinese Medicine Systems Pharmacology Database (tcmsp-e.com/index.php). Alkaloids with an OB value of ≥30% were selected for the next prediction and their structures were retrieved from the PubChem (pubchem.ncbi.nlm.nih.gov/) database. The Simplified Molecular Input Line Entry System (SMILES) representations of these alkaloids were subsequently imported into the Swiss Target Prediction database (swisstargetprediction.ch), where only targets with prediction probability scores >0.1 were retained as potential drug targets. Subsequently, the key words ‘non-small cell lung cancer’ were searched in the Genecards database (https://www.genecards.org) to identify relevant targets. Based on the binary relationships between NSCLC and its targets, a target network containing 5,388 nodes was constructed. Finally, the Cytoscape (cytoscape.org/, version 3.8.2) was applied to create a combined ‘drug-component-target-disease’ network diagram.

### Construction of a drug target PPI network

In total, 493 potential targets of 139 alkaloids and 5,387 NSCLC-related targets were imported into the Venny (bioinfogp.cnb.csic.es/tools/venny/, version 2.1.0) software, and the intersection of the two datasets was evaluated, which led to the identification of 304 common targets. These targets were then input into the STRING database (https://cn.string-db.org/) for analysis, with the organism restricted to ‘*Homo sapiens*’. The minimum interaction threshold was established at 0.4, resulting in the generation of target protein networks. The data were subsequently imported into Cytoscape (version 3.8.2) to construct a PPI network map.

### Screening for HUB genes

The PPI subnetwork was analyzed using the MCODE (version 2.0.3) clustering algorithm (apps.cytoscape.org/apps/mcode); Cytoscape (version 3.8.2) and the MCODE plug-in were used for network topology analysis with the following parameters: Degree cut-off=2, a node score cut-off=0.2, a K-core of 2 and a maximum depth=100. Network topology parameters (degree, betweenness and closeness centrality) for these targets were obtained using the Analysis Network tool in Cytoscape. The CytoNCA (version 2.1.6; cytoscape.org/apps/cytonca) and CytoHubba (version 0.1) plugins (apps.cytoscape.org/apps/cytohubba) were used to identify HUB genes. Finally, a Venn diagram was constructed to identify the overlapping targets.

### Functional enrichment analysis of the PPI core subnetwork

The core subnetworks derived from the clustering analysis of the PPI network were analyzed using the STRING database and DAVID databases (david.ncifcrf.gov/summary.jsp), Reactome (reactome.org/) and WikiPathways (wikipathways.org/) databases for the enrichment analysis (Gene Ontology (GO; geneontology.org/) and Kyoto Encyclopedia of Genes and Genomes (KEGG; kegg.jp/)). The enrichment results were organized by intensity, gene count (≥15) and P≤0.05, ranked from high to low scores, with the top 20 selected for visualization.

### Experimental materials and reagents

Imperialine, verticinone, verticine and peimisine were purchased from Chengdu Push Bio-Technology Co., Ltd., imperialine-3-β-D-glucoside and delavine were obtained from Chengdu Herbpurify Co., Ltd., and ebeiedinone and delavinone from Chengdu Must Bio-Technology Co., Ltd. The purity of all compounds was >98%. The Annexin V-FITC/propidium iodide (PI) apoptosis detection kit was acquired from Dojindo Laboratories, Inc. The BCA protein kit and RIPA buffer were from Beyotime Institute of Biotechnology and the protease inhibitor cocktail was from Roche Diagnostics. Phenylmethylsulfonyl fluoride and phosphatase inhibitor were purchased from Beijing Solarbio Science & Technology Co., Ltd. Chloroquine (CQ) and dimethyl sulfoxide were obtained from MilliporeSigma, and the Cell Counting Kit-8 (CCK-8) from Beijing Solarbio Science & Technology Co., Ltd. Antibodies specific for PARP (cat. no. CY5347) and cleaved PARP (cat. no. CY5265) were obtained from Shanghai Abways Biotechnology Co., Ltd.; antibodies for cleaved caspase-3 (cat. no. 9664T), LC3A/B (cat. no. 12741), GAPDH (cat. no. 2118) and HRP-conjugated goat anti-rabbit IgG secondary antibody (cat. no. ab6721) were obtained from Cell Signaling Technology, Inc. All other chemical reagents used were of analytical grade.

### NSCLC cell lines and cultures

In total, three non-small cell lung cancer cell lines, NCI-H1975 (CRL-5908), NCI-H1299 (CRL-5803) and A549 (CCL-185), were obtained from the American Type Culture Collection. The cell lines were maintained in RPMI-1640 medium supplemented with 10% (v/v) fetal bovine serum (FBS), 100 U/ml penicillin and 100 µg/ml streptomycin (all Gibco; Thermo Fisher Scientific, Inc. at 37°C in a 5% CO_2_ atmosphere, and the cell culture medium was replaced daily.

### Cell viability assay

A single-cell suspension in the logarithmic growth phase was prepared and counted. Subsequently, 100 µl of the single-cell suspension were added to each well of a 96-well plate, with a concentration of 4×10^3^ cells/well for the A549 cells and 5×10^3^ cells/well for the NCI-H1299 cells, diluted with complete medium. Control groups included blank control group, negative control group and experimental groups, with each group containing ≥4 replicate wells. Following 48 h of drug treatment, 10 µl CCK-8 regent were added to each well. The wells were then incubated in the dark for 4 h, and the absorbance was measured at 450 nm using a microplate reader (Bio-Rad Laboratories, Inc.).

### Annexin V-FITC/PI staining assay

Early and late apoptosis cells were evaluated using flow cytometry. In brief, NCI-H1299 and A549 cells were treated with ebeiedinone (10, 15, 20 and 30 µM), verticinone (15, 30 µM) and gefitinib (15, 30 µM) for 48 h in a 37°C incubator. A blank control without medication and a 5-fluorouracil group (15, 30 µM) were set up. 5-Fluorouracil was used as a positive control drug for apoptosis to determine whether cells can undergo apoptosis. Gefitinib is used as a positive drug for comparison with Verticinone and ebeiedinone, to determine the ability to induce apoptosis. For the flow cytometry experiments, the cells were stained with 5 µl annexin V-FITC and 5 µl PI at room temperature for 15 min. The fluorescence intensity was measured using a BD FACSCalibur flow cytometer (Becton Dickinson and Company), and the apoptotic rates were analyzed using the NOVAexpress software (version 1.6.2; Agilent).

### Western blot analysis

A549 cells were harvested and lysed using a nuclear and cytoplasmic protein extraction kit (Beyotime Biotechnology; cat. no. P0028,) according to the manufacturer's instructions. Protein concentrations were determined using the BCA protein assay kit. A total of 20 µg of protein sample to each well. Cytoplasmic proteins were separated by 7 and 15% SDS-PAGE and transferred to a polyvinylidene difluoride membrane (MilliporeSigma). The membranes were blocked with 5% skimmed milk at room temperature for 1 h, followed by incubation with primary antibodies at 4°C overnight. This was followed by incubation with goat anti-rabbit IgG (H&L) (cat. no. ab6721; 1:10,000; Abcam, Cambridge, UK) at 37°C for 2 h. Protein bands were visualized using the Immobilon Western HPR Substrate (MilliporeSigma) and images were captured with the GelView 6000Plus Chemiluminescence Imaging System (Guangdong Biolight Meditech Co., Ltd.). The Image J software (version 1.54; National Institutes of Health) was used for protein quantification. The following are the primary antibodies used in this experiment: GAPDH (14C10) Rabbit Monoclonal Antibody (cat. no. 2118; 1:1,000; Cell Signaling Technology); β-tubulin (9F3) Rabbit Monoclonal Antibody (cat. no. 2128; 1:1,000; Cell Signaling Technology, Inc.); PARP 1 Ab (cat. no. CY5347; 1:1,000); Cleaved PARP (cat. no. CY5265; 1:1,000; both Shanghai Abways Biotechnology Co., Ltd.); Cleaved caspase-3 (cat. no. 9664T; 1:1,000; Cell Signaling Technology), LC3A/B (D3U4C) Rabbit Monoclonal Antibody (cat. no. 12741; 1:1,000; Cell Signaling Technology; The United States). p62 (D1Q5S) Rabbit Monoclonal Antibody (cat. no. 39749; 1:1,000; Cell Signaling Technology; The United States)

### Statistical analysis

All data are presented as the mean ± standard deviation. All statistical analyses were performed using GraphPad Prism software (version 7.0; Dotmatics). Statistical analyses were performed as follows: a one-way ANOVA was used for multi-group comparisons, with Tukey's HSD post hoc test applied for further pairwise analysis. The Student's t-test (unpaired) was used for direct comparisons between two groups. P<0.05 was considered to indicate a statistically significant difference.

## Results

### Alkaloid-NSCLC biological target network

**Figure 1.**
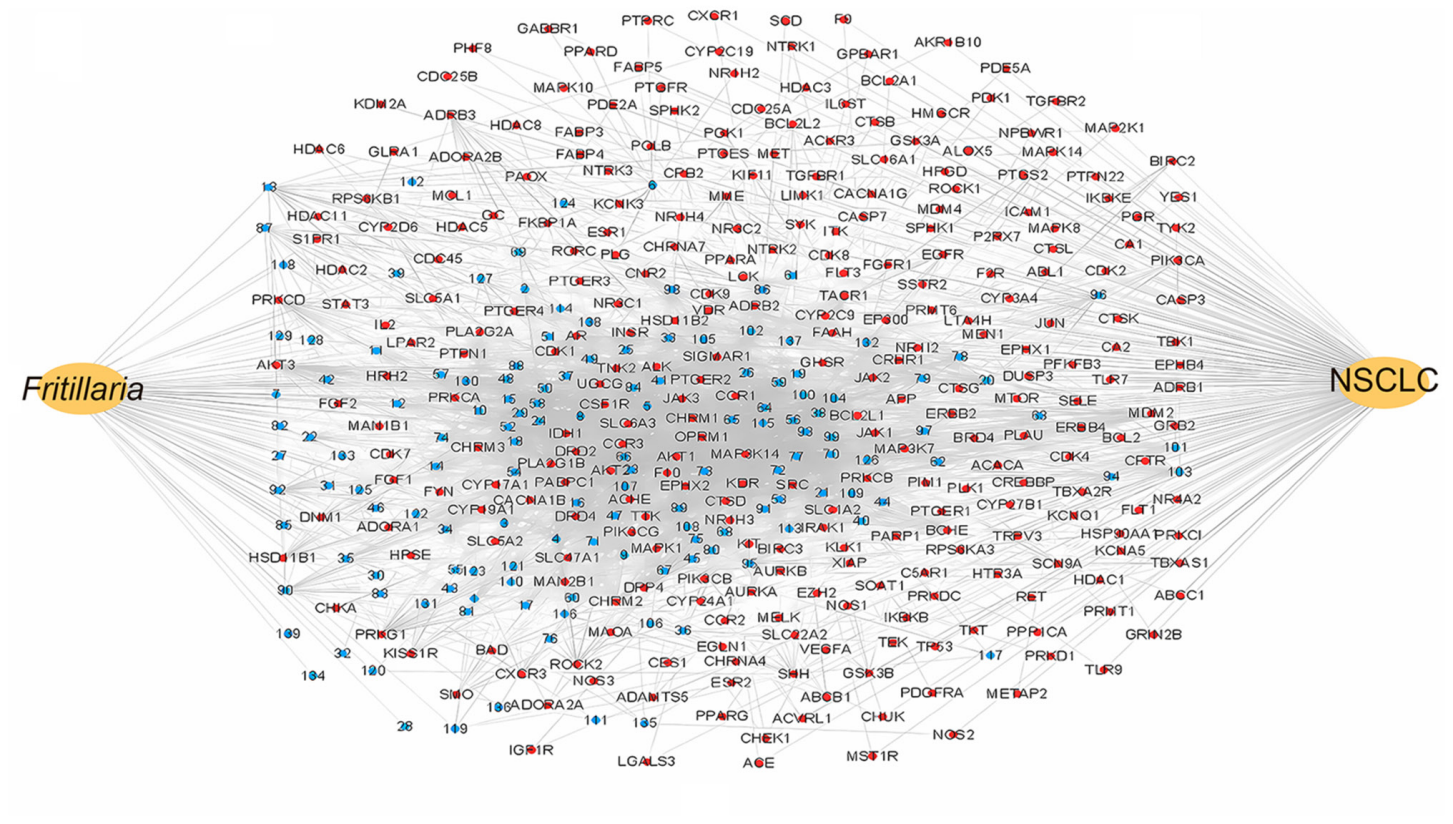
Target network of alkaloids in *Fritillaria.* Alkaloids components are depicted in blue and targets in red. NSCLC, non-small cell lung cancer.

Following a comprehensive literature search and retrieval of data from PubChem records, the SMILES representations of alkaloids in *Fritillaria* were imported into the Swiss Target Prediction program to identify drug targets that met the specified criteria (Table SI). Target genes for NSCLC were obtained following retrieval from the GeneCards database. These data were integrated to construct a ‘drug-component-target-disease’ network using Cytoscape (Fig. 1). The degree-based ranking of the top 20 pharmacologically active compounds is summarized in Table I.

### Drug target PPI network screening for HUB genes

**Figure 2.**
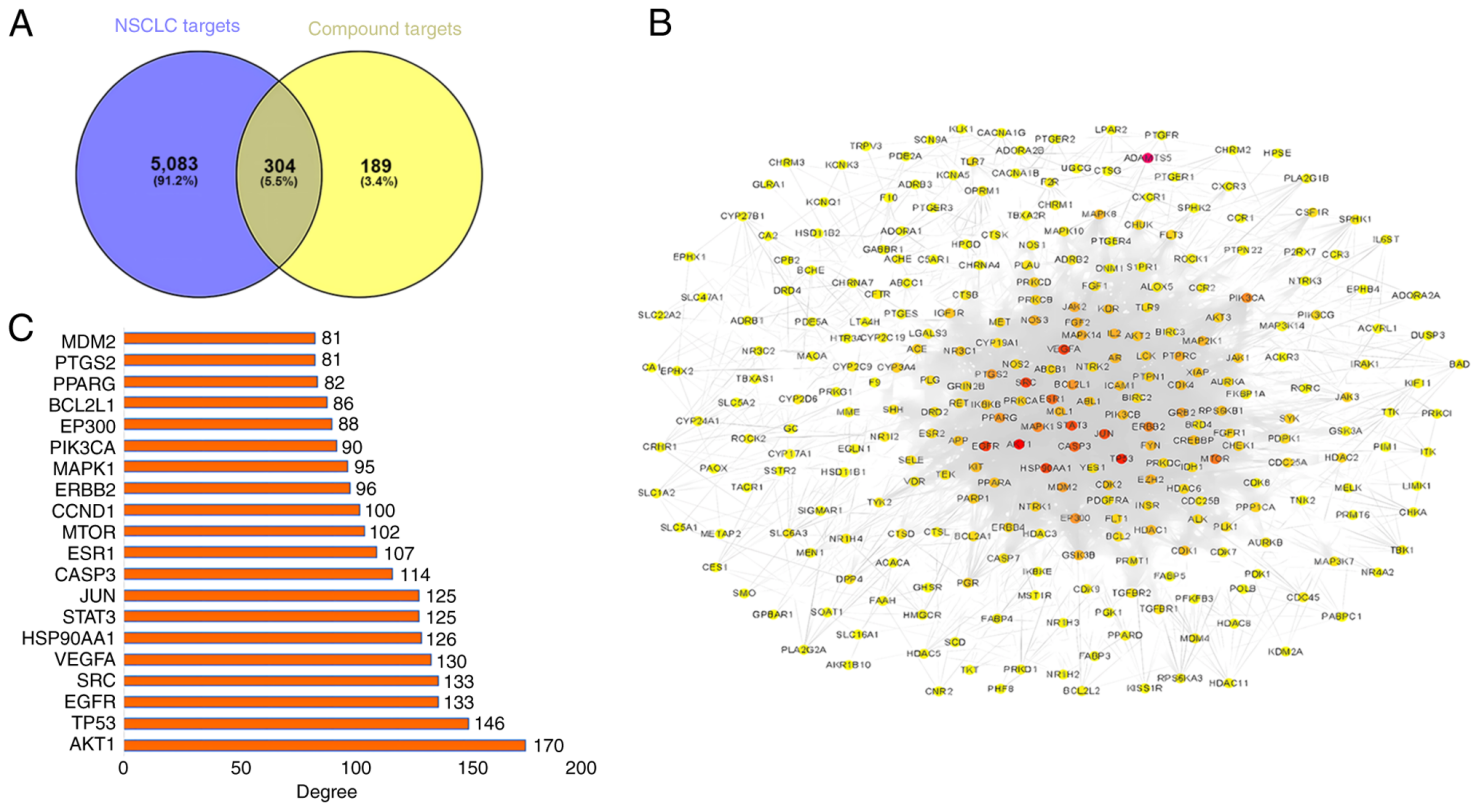
Target networks of alkaloids and NSCLC. (A) Intersection of the NSCLC target and alkaloid target sets. (B) NSCLC target PPI network of alkaloids in *Fritillaria.* (C) Top 20 core targets in the PPI network of NSCLC proteins targeted by alkaloids. NSCLC, non-small cell lung cancer; PPI, protein-protein interaction.

A Venn diagram (Fig. 2A) was generated using Venny (version 2.1.0) to identify the intersection between 493 potential targets from 139 alkaloids in *Fritillaria* and 5,387 disease targets associated with NSCLC. In total, 304 common targets were identified within the NSCLC-related and potential alkaloid target sets. These common targets were subsequently input into the STRING database to retrieve target protein networks, and the results were imported into the Cytoscape software (version 3.8.2) to create a drug target PPI network diagram (Fig. 2B). Cytoscape (version 3.8.2) and the MCODE plug-in were used to analyze the topology of the PPI network. The results revealed several targets with high degree values within this PPI network, indicating a greater number of interactions with other proteins. The top 20 targets, with degree values ranging from 80 to 170, are illustrated in Fig. 2C.

**Figure 3.**
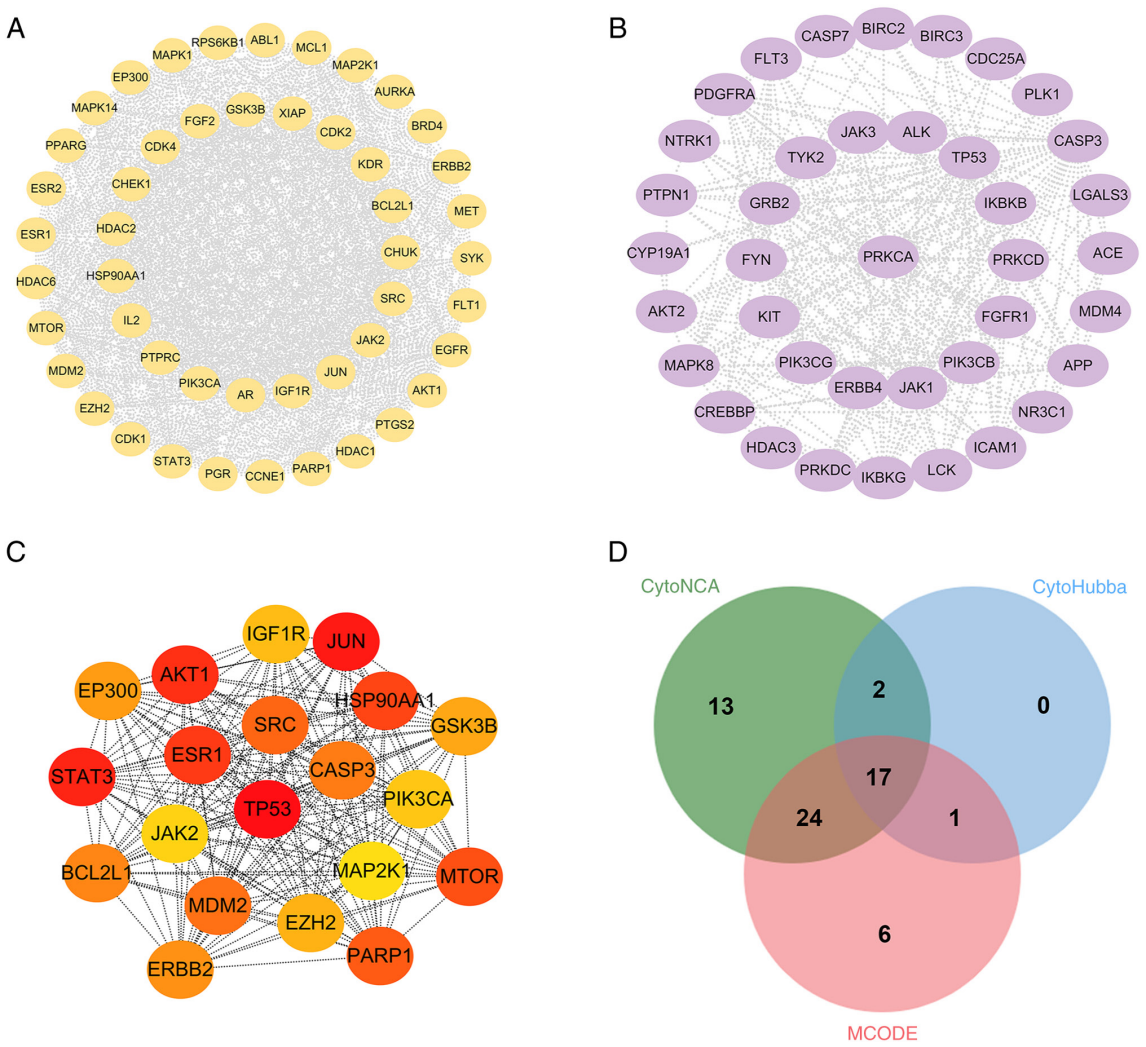
Identification of hub genes. (A, B) The two protein clusters obtained in the MCODE plug-in analysis. (C) Top 20 proteins ranked by score following cluster analysis of the PPI network using CytoHubba. (D) Venn diagram of CytoNCA, Cytohubba and MCODE targets. NSCLC, non-small cell lung cancer.

In the MCODE cluster analysis, the PPI network was divided into 11 subnetworks (Table II), of which four contained >10 nodes (Fig. 3A and B). In addition, further CytoHubba analysis was performed on the PPI network, yielding the top 20 targets with the highest scores (Fig. 3C) and CytoNCA analysis was also conducted. Finally, the intersection of the enriched Cluster 1, CytoNCA and CytoHubba-related targets was identified using a Venn diagram, yielding 17 common targets (Fig. 3D), namely: MDM2, EP300, PIK3CA, STAT3, ERBB2, EZH2, GSK3B, HSP90AA1, MTOR, PARP1, JUN, AKT1, IGF1R, ESR1, SRC, BCL2L1 and JAK2. These targets are associated with tumor apoptosis, tumor invasion and immune evasion (26–29). Among these, BCL2 is a typical anti-apoptotic protein in the apoptotic pathway, providing insights for subsequent experimental validation (30).

### Functional enrichment analysis

**Figure 4.**
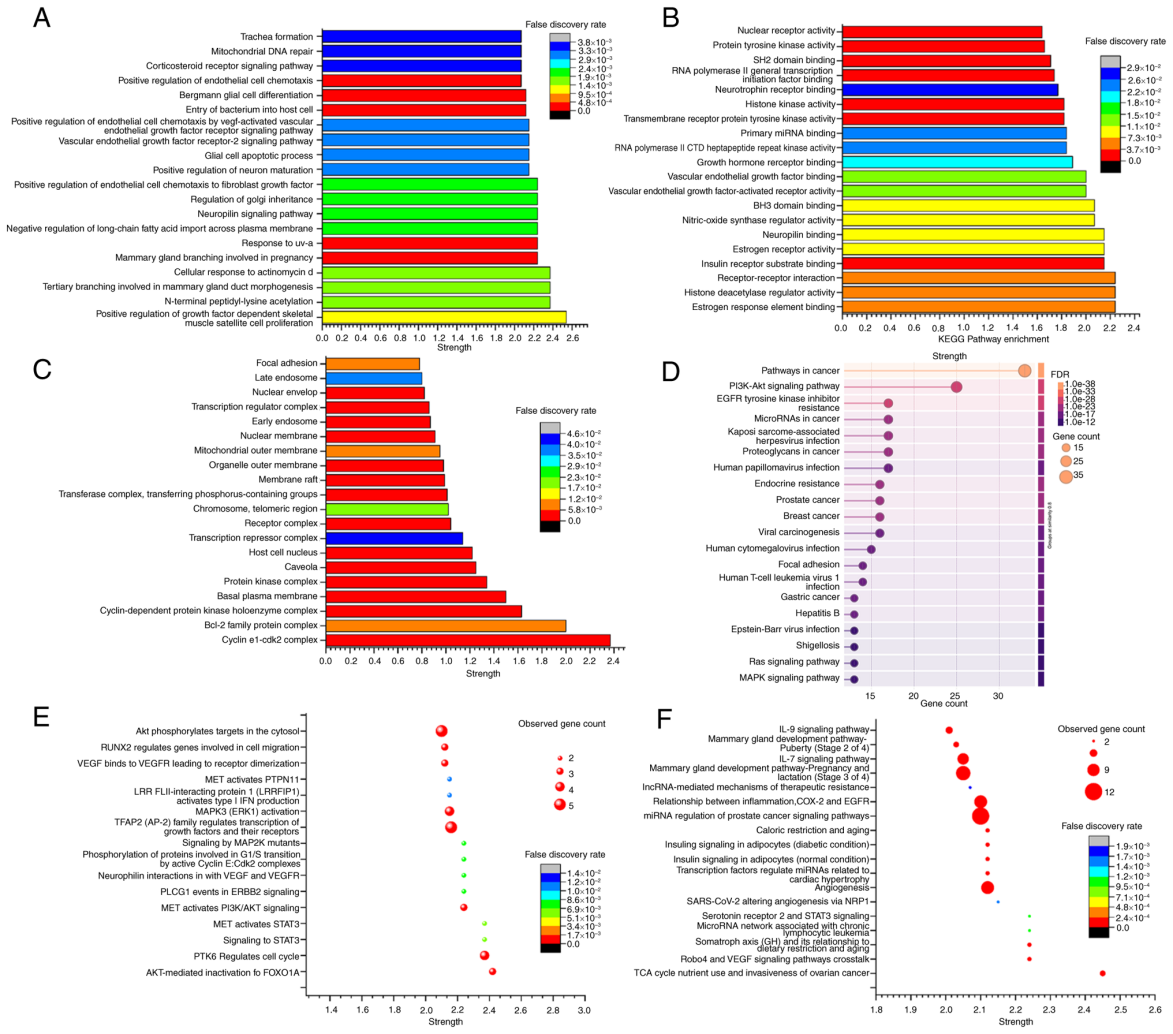
Visualization of the top 20 enrichment intensities of cluster 1 using various methods. (A) GO biological processes. (B) GO molecular functions. (C) GO cellular components. (D) KEGG pathway enrichment. (E) Reactome pathway enrichment. (F) WikiPathways enrichment. The enrichment results were organized by intensity, gene count (≥15) and P-value (≤0.05), ranked from high to low scores, with the top 20 selected for visualization.

The core subnetworks derived from the PPI network clustering analysis were examined using the STRING database for enrichment using GO, KEGG, Reactome and WikiPathways (Fig. 4). The results were organized in descending order based on the enrichment intensity and P-value.

In the GO enrichment analysis, cluster 1 was found to be enriched in 1,026 biological process terms and 104 molecular function terms (P≤0.05). Additionally, a total of 58 cellular component terms and the top 20 enriched pathways are presented in Fig. 4A-C. KEGG pathway enrichment analysis revealed the enrichment of 151 pathways in cluster 1, showing association with ‘Pathways in cancer’, ‘PI3K-Akt signaling pathway’, ‘EGFR tyrosine kinase inhibitor resistance’ and other tumor-related pathways ranked highest in the enrichment index (Fig. 4D).

Reactome pathway enrichment analysis demonstrated the enrichment of 281 pathways in cluster 1, with numerous tumor-associated pathways ranked highly in the enrichment index. These include pathways related to ‘AKT-mediated inactivation of FOXO1A’, ‘PTK6 Regulates cell cycle’, ‘signalling to STAT3’, ‘MET activated STAT3’ and other tumor signal transduction pathways (Fig. 4E). The WikiPathway enrichment analysis revealed the enrichment of 263 pathways in cluster 1. Among these, the ‘Robo4 and VEGF signaling pathways crosstalk’, ‘TCA cycle nutrient use and invasiveness of ovarian cancer’, ‘serotonin receptor 2 and STAT3 signaling’ and various other tumor-related pathways were highly ranked (Fig. 4F).

Based on the results of pathway enrichment analysis using different methods, it was preliminarily hypothesized that the active components in *Fritillaria cirrhosa* may exert certain effects on NSCLC. Combined with the results of core target enrichment analysis, it was considered that these effects may be achieved by promoting tumor cell apoptosis and autophagy and inhibiting immune escape, providing a basis for further verification.

### Anti-proliferative ability of isosteroid alkaloids

**Figure 5.**
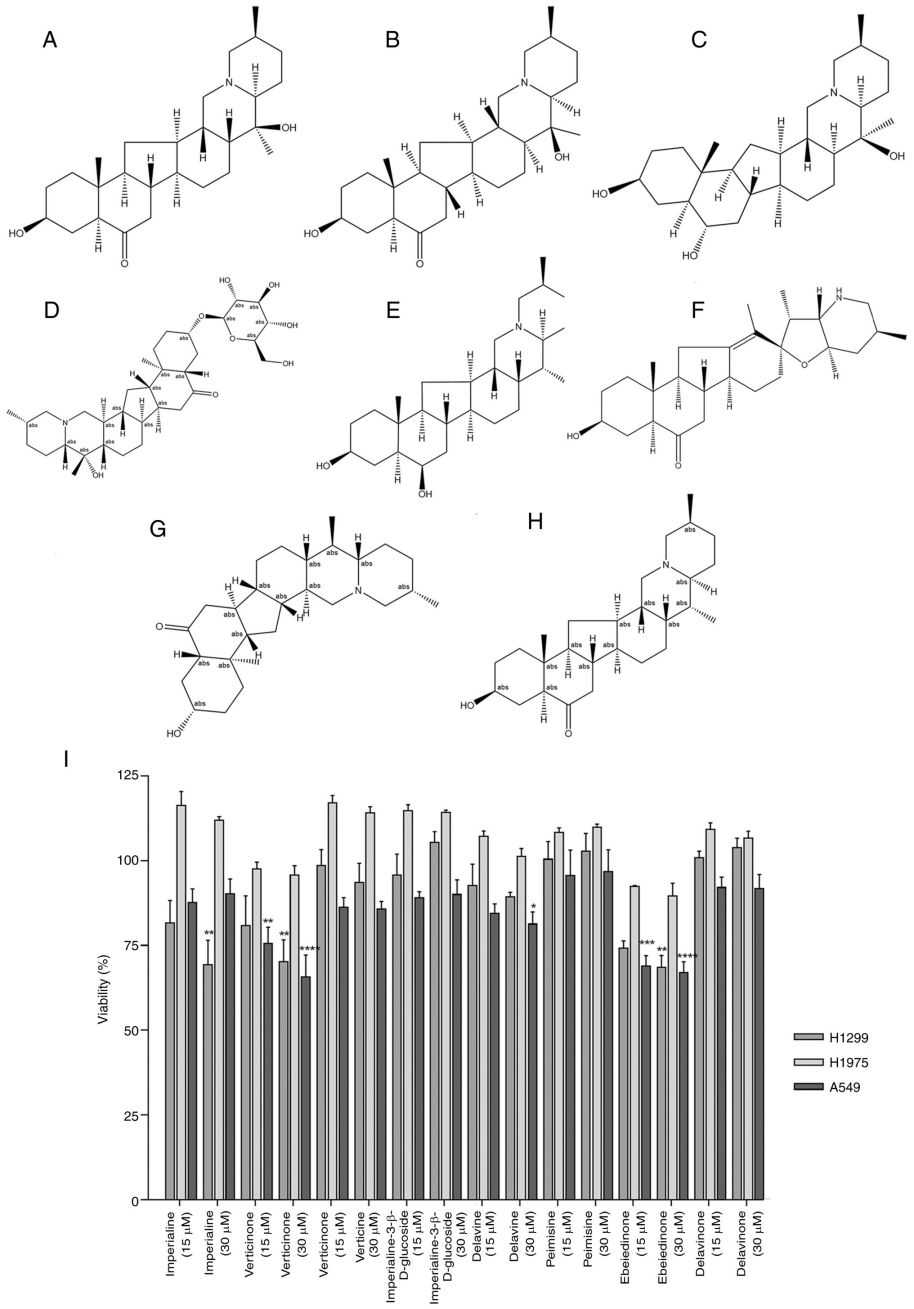
Structures of isosteroid alkaloids and their anti-proliferative effects of NSCLC cells. (A) Imperialine, (B) verticinone, (C) verticine, (D) imperialine-3-β-d-glucoside, (E) delavine, (F) peimisine, (G) ebeiedinone and (H) delavinone. (I) Effects of isosteroid alkaloids on the viability of three different cell lines, H1299, H1975 and A549. Cells were treated with alkaloids at doses of 15 and 30 µM for 48 h. *P<0.05, **P<0.01, ***P<0.001, ****P<0.0001 vs. control. NSCLC, non-small cell lung cancer.

The enrichment analysis suggested that the anticancer activity of alkaloids is closely related to the regulation of autophagy and apoptosis. Previous reports have indicated that alkaloids exert their anticancer effects through anti-inflammatory feedback (31,32). Furthermore, our previous research has shown that imperialine, verticinone, verticine, imperialine-3-β-D-glucoside, delavine, and peimisine can inhibit cigarette-induced oxidative stress and inflammatory responses in RAW264.7 cells (33). Based on the aforementioned network pharmacology results, this study ultimately selected imperialine, verticinone, verticine, imperialine-3-β-D-glucoside, delavine, peimisine, ebeiedinone, and delavinone as the subjects of investigation (Fig. 5). The effects of these eight distinct isosteroid alkaloids (Fig. 5A-H) derived from *Fritillaria* on the viability of three types of NSCLC cells were evaluated using CCK-8 assay. No significant effects on the viability of NCI-H1975 cells were observed for the majority of the alkaloids (Fig. 5I). By contrast, the survival rates of the NCI-H1299 and A549 cells treated with verticinone and ebeiedinone decreased to 75.57±4.80% and 68.9±3.06% (at 15 µM), and 65.75±6.44% and 67±3.20% (at 30 µM), respectively (Table SII, Table SII, Table SIII, Table SIV). Based on these findings, verticinone and ebeiedinone were selected for further analyses.

### Induction of apoptosis by isosteroidal alkaloids

**Figure 6.**
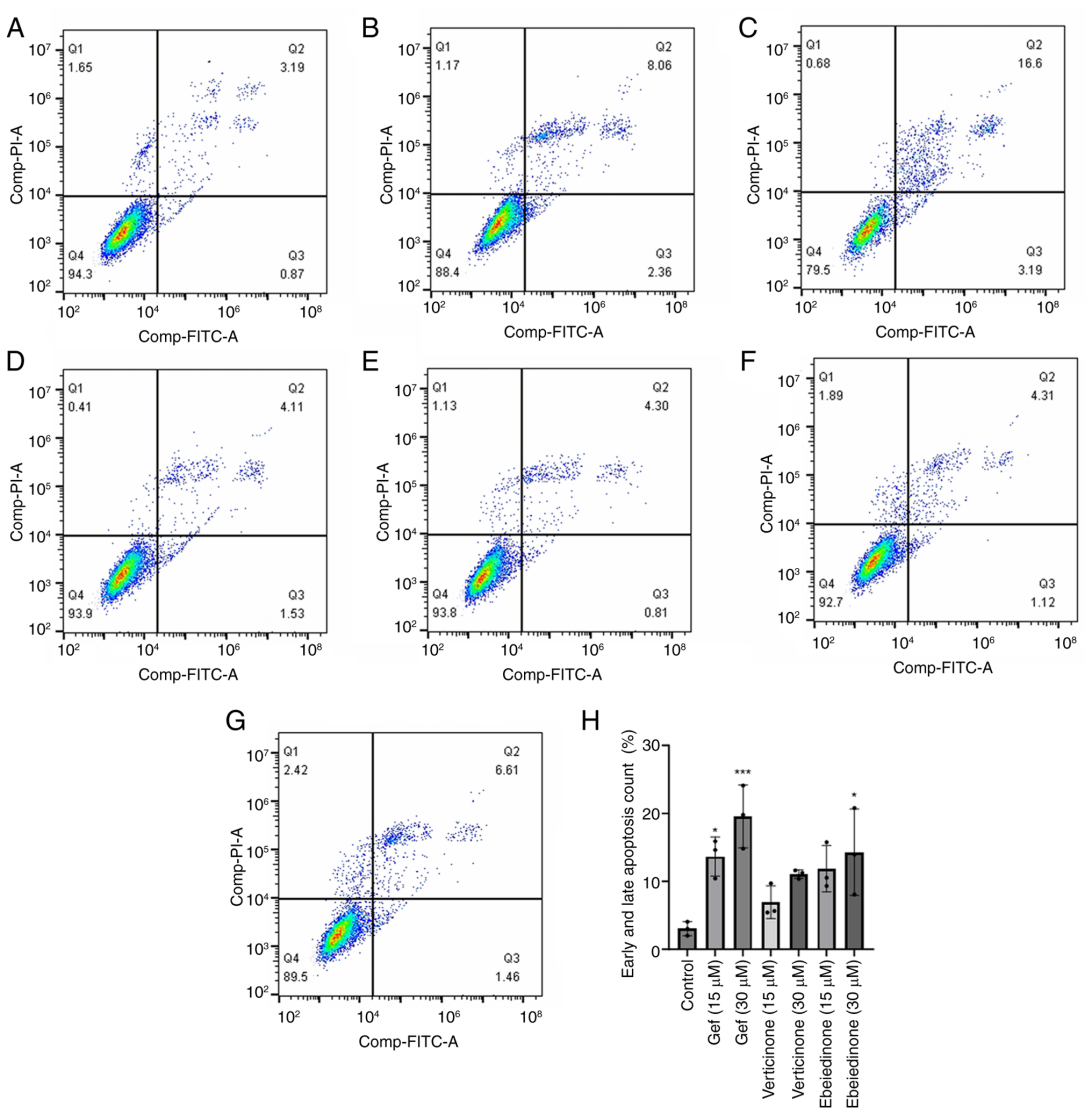
Effects of verticinone and ebeiedinone on apoptosis in NCI-H1299 cells following 48 h of treatment. (A) Negative control group. Gefitinib positive control (B) 15 µM). (C) Gefitinib positive control groups (30 µM). (D) Verticinone treatment groups (15 µM). (E) Verticinone treatment groups (30 µM). (F) Ebeiedinone treatment groups (15 µM). (G) Ebeiedinone treatment groups (30 µM). (H) Quantitative results of NCI-H1299 cell apoptosis. ^*^P<0.05, ***P<0.001 vs. control.

**Figure 7.**
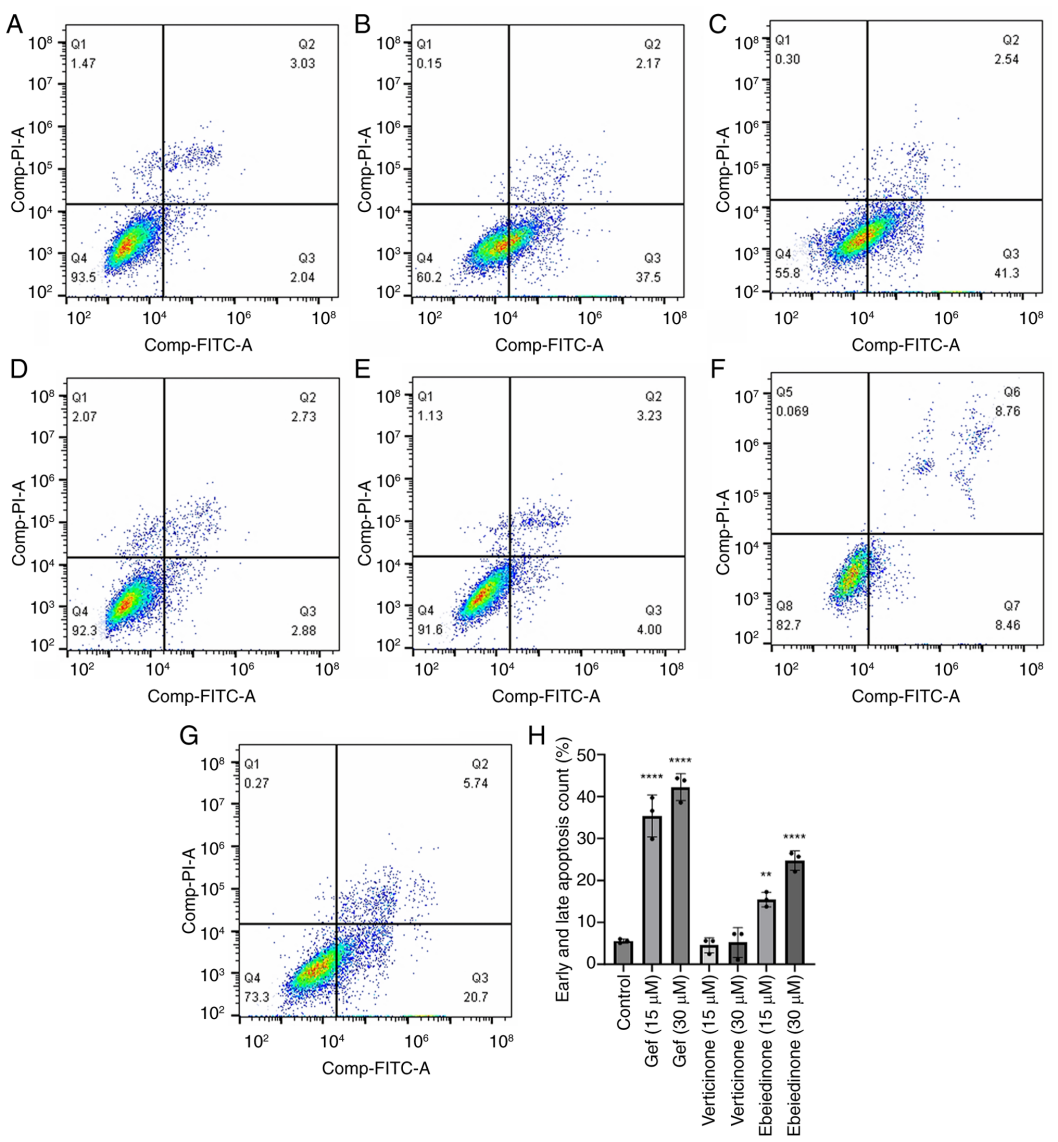
Effects of verticinone and ebeiedinone on A549 cells apoptosis following 48 h of treatment. (A) Negative control group. Gefitinib positive control (B) (15 µM). (C) Gefitinib positive control groups (30 µM). (D) Verticinone treatment groups (15 µM). (E) Verticinone treatment groups (30 µM). (F) Ebeiedinone treatment groups (15 µM). (G) Ebeiedinone treatment groups (30 µM). (H) Quantitative results of A549 cell apoptosis. ^**^P<0.01, ****P<0.0001 vs. control.

**Figure 8.**
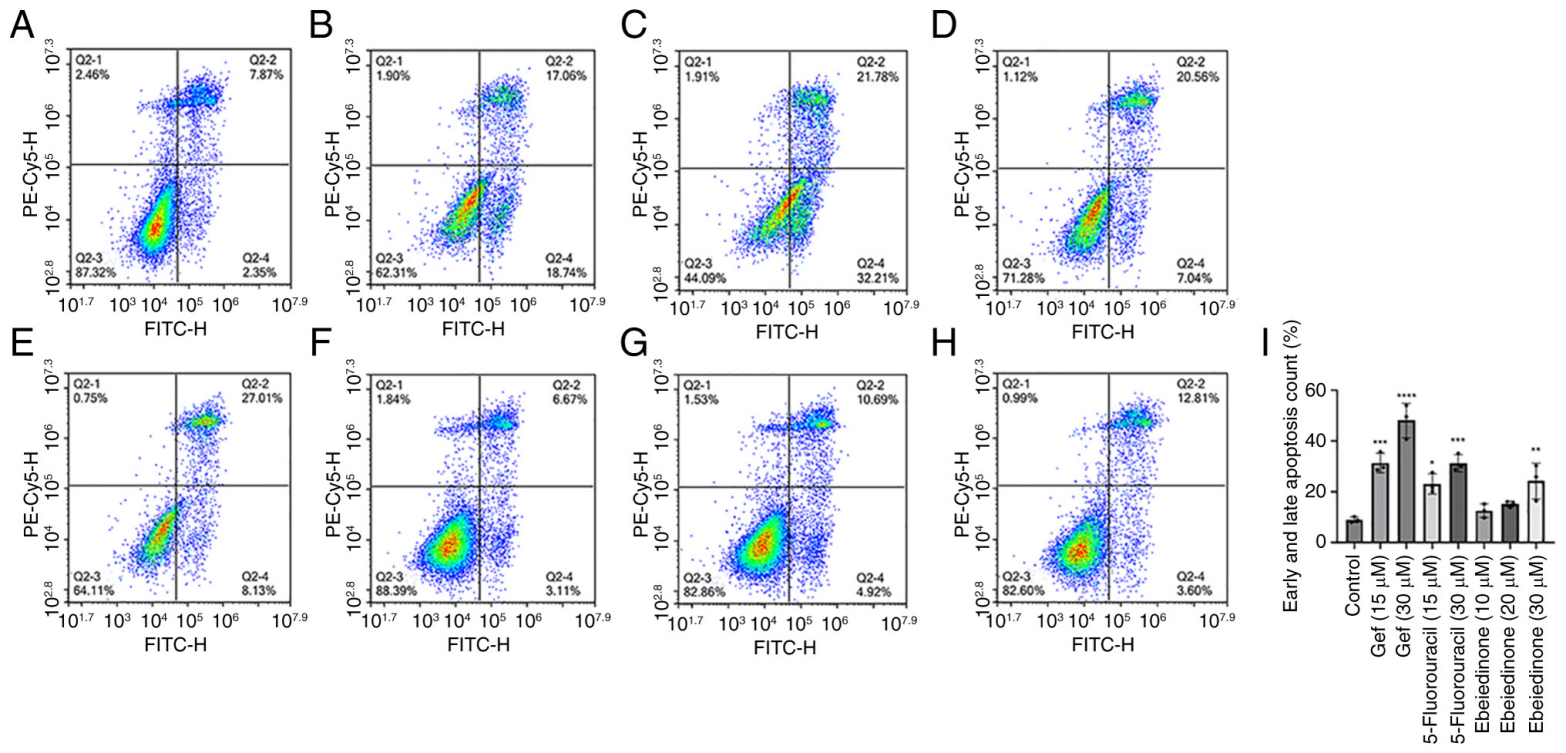
Effects of different doses of ebeiedinone on apoptosis in A549 cells following 48 h of treatment. (A) Negative control group. (B) Gefitinib positive control groups (15 µM). (C) Gefitinib positive control groups (30 µM). (D) 5-Fluorouracil treatment groups (15 µM). (E) 5-Fluorouracil treatment groups (30 µM). (F) Ebeiedinone treatment groups (10 µM). (G) Ebeiedinone treatment groups (20 µM ). (H) Ebeiedinone treatment groups (30 µM). (I) Quantitative results of A549 cell apoptosis. *P<0.05, **P<0.01, ***P<0.001, ****P<0.0001 vs. control.

To investigate the potential mechanisms underlying the alkaloid-induced decrease in cell viability, cell death was detected using the Annexin V-FITC/PI dual labeling assay. The number of Annexin V-FITC-positive NCI-H1299 cells was comparable between the groups (Fig. 6). The group treated with gefitinib exhibited rates of 10.42 and 19.79% at concentrations of 15 and 30 µM, respectively (Fig. 6A-G). The number of Annexin V-FITC-positive A549 cells increased following treatment with ebeiedinone (Fig. 7A-G). Subsequently, experiments with a range of ebeiedinone concentrations were conducted. The results indicated that ebeiedinone promoted apoptosis in a concentration-dependent manner (Fig. 8A-H). Furthermore, analysis revealed that cells treated with dehydro-eudobelin (30 μM) exhibited significant differences compared to the blank control group, indicating that dehydro-eudobelin has the ability to induce apoptosis (Fig. 8I).

### Effects of ebeiedinone on apoptosis- and autophagy-related protein expression in A549 cells

**Figure 9.**
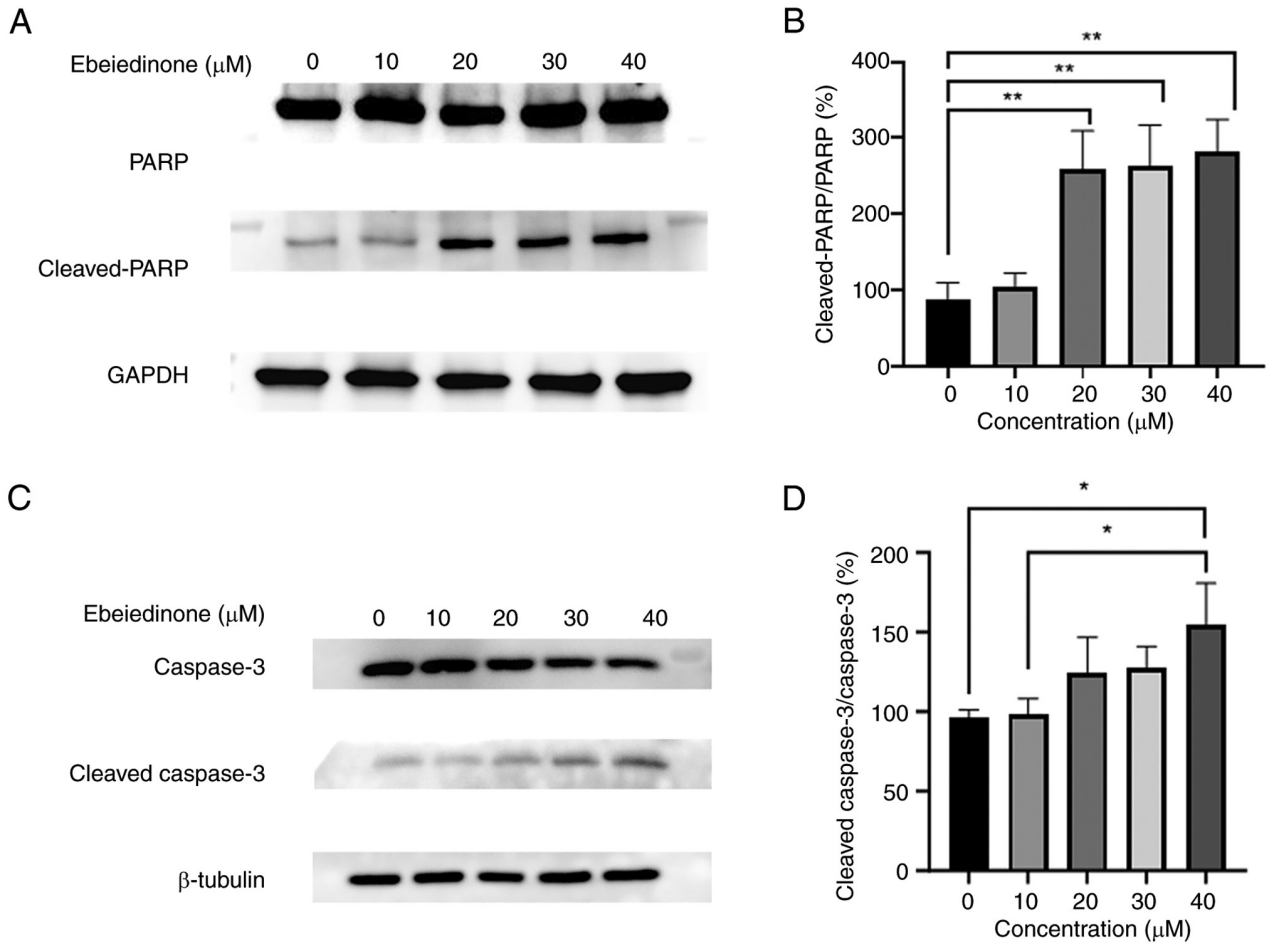
Increasing doses of ebeiedinone promoted cell apoptosis of NSCLC cells. (A) A549 cells were incubated with ebeiedinone (10, 20, 30 and 40 µM) for 48 h, and then the protein expression levels of PARP and cleaved PARP were examined via western blotting. (B) Greyscale analysis on protein expression cleaved PARP/PARP in A549 cells following 48 h of treatment. (C) The expression of cleaved caspase-3. (D) Protein expression cleaved caspase 3/ caspase 3 in A549 cells following 48 h of treatment. *P<0.05, **P<0.01 vs. control. NSCLC, non-small cell lung cancer.

Apoptosis is a key biological process in multicellular organisms, characterized in part by the activation of caspases and the induction of caspase-dependent cell death. Caspases are regarded as the central mediators of apoptosis (34). PARP, along with genomic stability factors and DNA repair enzymes, was originally identified as a key proteolytic substrate that is degraded by caspase-3 and other cysteine proteases during apoptosis (35). Increased levels of cleaved PARP and cleaved caspase-3 in the cells exposed to ebeiedinone were demonstrated in the present study (Fig. 9). The increase in the level of cleaved caspase-3 was observed across various drug concentrations and was comparable to the effect induced by the positive control drug, gefitinib (Fig. 9A and C). Protein expression levels of cleaved PARP/PARP and cleaved capase-3/GAPDH significantly increased (Fig. 9B and D). In summary, these findings demonstrated that ebeiedinone significantly stimulated the apoptosis of NSCLC cells.

**Figure 10.**
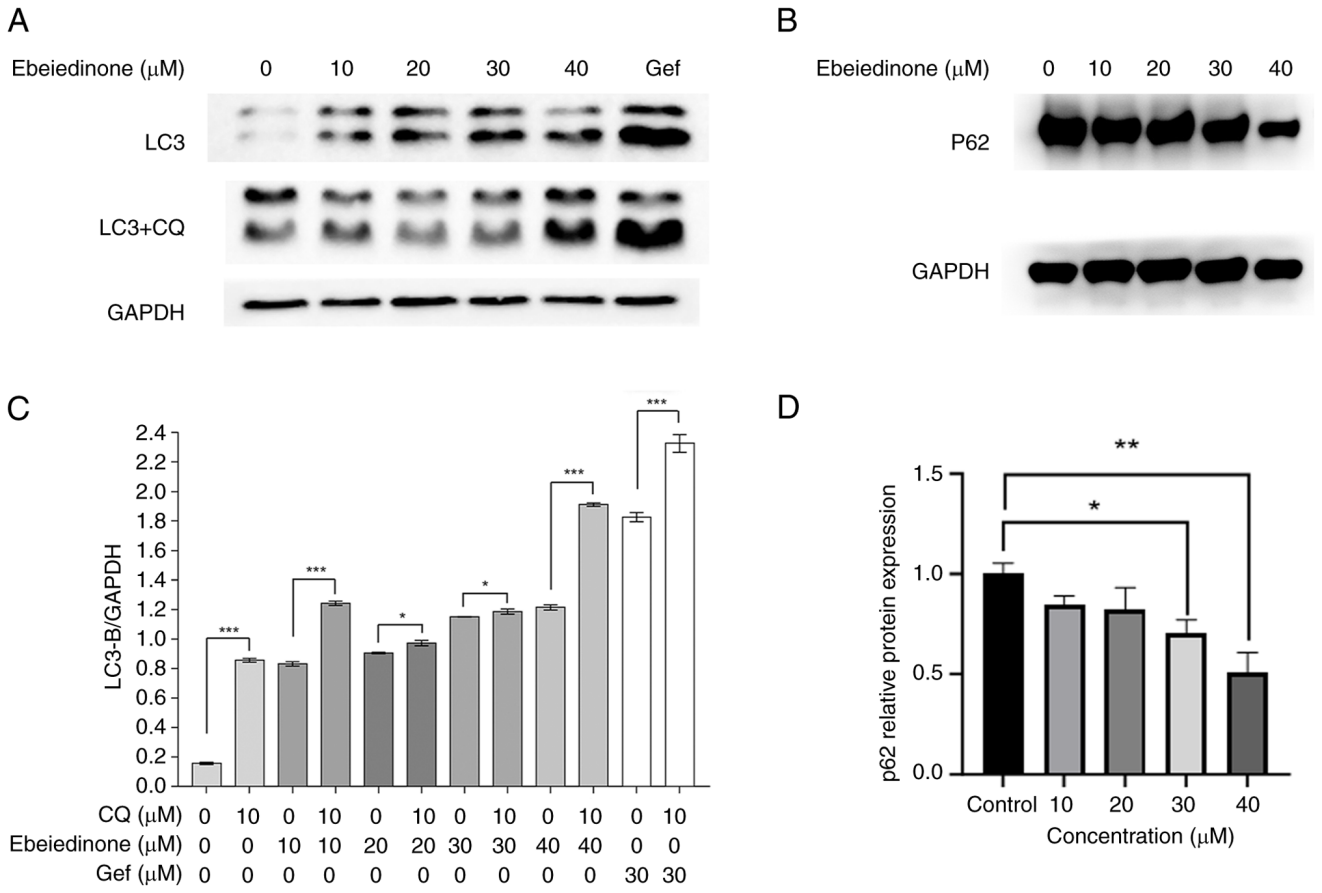
A549 cells were pre-treated with or without CQ (10 µM, 1 h). (A,B) The effects of different doses of ebeiedinone (10, 20, 30 and 40 µM) on autophagy markers after 48 h of treatment were determined via western blot. Positive control group (Gef): 30 µM. (C) Protein expression of LC3-II/GAPDH in A549 cells following 48 h of treatment. (D) Greyscale analysis on protein expression of p62 in A549 cells following 48 h treatment. *P<0.05, **P<0.01, ***P<0.001 vs. control. CQ, chloroquine; Gef, gefitinib.

Subsequently, it was investigated whether ebeiedinone induces autophagy in A549 cells by examining the expression of LC3-II, a protein marker of autophagy (Fig. 10). Ebeiedinone induced LC3-II protein expression in a concentration-dependent manner in A549 cells. Furthermore, when ebeiedinone was used in combination with the autophagy inhibitor, CQ (10 µM, 1 h), which disrupts lysosomal function to block autophagy (36), LC3-II expression was further upregulated (Fig. 10A and C). Additionally, the effects of ebeiedinone on p62 protein expression levels were assessed, which demonstrated that the ebeiedinone reduces p62 expression, thereby promoting cellular autophagy (Fig. 10B and D). These results further indicated that ebeiedinone could promote autophagy in A549 cells.

## Discussion

The present network-based pharmacology analysis suggested that the therapeutic activity of alkaloids in *Fritillaria* against NSCLC involves the interactions of >300 proteins. The alkaloids may exert antitumor effects by influencing various signaling pathways and interfering with tumor cell components, molecular functions and biological processes. The present study also collected relevant core targets such as MDM2, EP300, PIK3CA and STAT3 through cluster analysis, and found that these targets might be related to apoptosis and autophagy. Pathway enrichment analysis of this study, it was found that the alkaloids in *Fritillaria* might affect NSCLC through the PI3K-Akt signaling pathway.

PI3K-Akt signaling pathway is an important pathway for cell apoptosis and autophagy. In numerous types of human cancer, the constitutively active oncoprotein, AKT1, suppresses autophagy (37). In lung cancer, the inhibition of AKT is also strongly associated with increased apoptosis and autophagy. Luo *et al.* (38) discovered that SLL-1A-16 can induce autophagy in NSCLC cells by inhibiting the Akt/mTOR pathway, and simultaneously upregulate the expression of caspase-3 and Bax, significantly inducing cell apoptosis. Mechanistically, apoptosis is a complex, multi-step process primarily driven by a caspase-dependent proteolytic cleavage cascade. Akt promotes cell survival by blocking pro-apoptotic proteins. Akt negatively regulates the function and expression of Bcl-2 homology domain 3 (BH3) proteins, which inactivate members of the Bcl-2 family by binding to them. As a result, caspase-9, a downstream protein of Bcl-2, cannot be cleaved, thereby inhibiting the subsequent cascade of events and preventing cell apoptosis (39). Furthermore, the PI3K/Akt signaling pathway is also the core pathway of autophagy (40). The inhibition of Akt can further suppress the expression of mTOR, thereby promoting the increase of LC3, while P62 is degraded, and eventually autophagy occurs in the cell. Previous studies (33) have shown that certain alkaloids in *Fritillaria cirrhosa* reduce alleviate oxidative stress and inflammation in RAW264.7 macrophages. Since macrophages serve as the primary immune cells in the tumor microenvironment, their oxidative stress and inflammatory responses are key to polarization. Therefore, it was hypothesize that the aforementioned alkaloids (imperialine, verticinone, verticine, imperialine-3-β-D-glucoside, delavine, peimisine, ebeiedinone, and delavinone) may also possess certain antitumor effects. Concurrently, our previous work also demonstrated that ebeiedinone exerts a protective effect on human bronchial epithelial cells (BESA-2B) (41). Zhang *et al* (42) found that delavinone exhibits anti-colonic cancer activity. In summary, the results of preliminary experiments, literature reviews, and network pharmacology analyses suggest that the alkaloids in *Fritillaria* may induce apoptosis and autophagy, thus providing a foundation for subsequent *in vitro* validation. Notably, in the present study, the H1299 cells did not undergo apoptosis, even upon treatment with concentrations that induced significant cytotoxicity. This finding may be attributed to two possible factors. Firstly, H1299 is a cell line devoid of the p53 gene, which is highly associated with various types of human tumors. The deletion of p53 may therefore reduce the sensitivity of cells to initiation of the apoptotic program (15,43). Secondly, the inhibition of H1299 cell proliferation by isosteroidal alkaloids may not involve apoptosis and instead, may occur through pyroptosis or iron-triggered cell death. Further research is warranted to determine the underlying reasons and associated mechanisms.

The present study primarily investigated the *in vitro* antitumor activity of two bioactive alkaloids; however, their *in vivo* pharmacokinetics and mechanisms remain insufficiently characterized. In previous research, Wu *et al* (44) and Jin *et al* (45) independently conducted *in vivo* pharmacokinetic analyses of verticinone and ebeiedinone, respectively. Wu *et al* employed liquid chromatography-mass spectrometry (LC-MS) to characterize the pharmacokinetic profile of verticinone in rats, including its plasma protein binding rate *in vitro.* Both oral and intravenous bioavailability were markedly higher in male rats (oral bioavailability, 45.8% in males vs. 2.74% in females). Tissue distribution analysis revealed extensive penetration, with the highest affinity observed in intestinal tissue (intestine > stomach > liver > kidney > spleen > lung > heart > gonads > muscle > skin > fat > brain). Notably, verticinone exhibited high plasma protein binding rates across species (89.8–91.3% in rats vs. 89.5–94.6% in humans), suggesting negligible interspecies variability. These findings collectively indicate that first-pass metabolism critically governs verticinone pharmacokinetics, leading to sex-dependent exposure in rodents. Jin *et al* developed a validated ultra-performance LC-MS/MS method to evaluate ebeiedinone pharmacokinetics in mice. Their data revealed concentration-dependent oral bioavailability, with an average absolute bioavailability of 30.60%. Prolonged mean residence time and elimination half-life following oral administration compared to intravenous injection further suggested substantial first-pass effects.

Furthermore, as a bioactive isosteroidal alkaloid derived from *F. cirrhosae* bulbus (a traditional Chinese medicinal herb), ebeiedinone demonstrates a lower pro-apoptotic efficacy compared with conventional agents such as cisplatin or paclitaxel. This observation aligns with the characteristic multi-targeted, gradual modulation of apoptotic pathways often seen with phytochemicals.

For example, a study by Li *et al* (46) demonstrated that total alkaloids from *Stephania tetrandra* (TAS) could induce apoptosis in A549 and H1299 cells. However, at concentrations far below the IC_50_, the proportions of early and late apoptotic cells showed no significant difference compared with that of the control group. This indicates that while TAS possesses pro-apoptotic activity, its effect is not pronounced at low doses. Notably, it was found that TAS could enhance the sensitivity of NSCLC cells to cisplatin and exert a synergistic effect with cisplatin to collectively increase apoptosis. Although that study did not delve deeper into the underlying mechanism, it provides a new perspective for the present research. Furthermore, it suggests that the limited direct apoptotic capacity of some natural products might reveal their potential utility in combination therapies.

The sub-IC_50_ dosing regimen employed in the present study was deliberately selected to elucidate mechanistic nuances, particularly the compound's concentration-dependent modulation of apoptosis-related proteins, rather than to elicit maximal cytotoxicity. Notably, western blotting demonstrated the dose-responsive upregulation of pro-apoptotic markers upon ebeiedinone treatment, thereby reinforcing its apoptotic induction capacity despite the limited Annexin V/PI signal amplitude. Moderated apoptotic effects may reflect polypharmacological profile of ebeiedinone, which likely engages parallel cytoprotective pathways to attenuate collateral damage. Future studies will explore structural optimization and synergistic combinations to amplify therapeutic potential of ebeiedinone while preserving the favorable safety trajectory. However, the present study had certain limitations. It is limited to *in vitro* studies of the anti-NSCLC efficacy at the cellular level and does not investigate its *in vivo* efficacy. Furthermore, this study did not delve into the specific mechanisms by which alkaloids exert their anti-NSCLC effects.

The aforementioned studies provide insight into the pharmacokinetic behavior of verticinone and ebeiedinone, establishing a framework for future pharmacodynamic and mechanistic investigations of these alkaloids *in vivo.* To the best of our knowledge, the present study is the first to demonstrate that ebeiedinone combined with CQ further increases the levels of LC3-II to promote the autophagic flux in NSCLC cells. The preliminary findings of the present study confirm the stimulatory effects of isosteroidal alkaloids in the bulbus *Fritillaria cirrhosa* on autophagy and apoptosis in A549 cells, thus warranting further research.

For the treatment of NSCLC, platinum-based chemotherapy remains the primary first-line therapy. However, it has a poor prognosis for metastatic NSCLC and is associated with severe toxic side effects, such as hearing loss (47,48). Bispecific antibody drugs are mainstream treatments in the therapeutic strategy for NSCLC; for example, amivantamab can simultaneously target both EGFR and MET (48). Nevertheless, these bispecific antibody drugs often face challenges such as high production costs due to manufacturing difficulties, risks of immunogenicity, and severe side effects caused by cytokine release syndrome (CRS) (49). Although ADC-type small-molecule drugs are a hot research topic for NSCLC treatment, they currently pose potential risks to *in vivo* biosafety (50). Therefore, identifying new drugs with good efficacy and low toxicity is a key focus of NSCLC research. Natural products isolated from medicinal plants represent a rich reservoir for anti-NSCLC agents. Increasing research has demonstrated that natural products possess potent anti-NSCLC activity and are being applied in clinical treatment (51,52). Ebeiedinone, discovered in this study, warrants further investigation into its mechanism of action and holds promise for future application as a novel drug in the clinical treatment of NSCLC.

## Supplementary Material

Supporting Data

## Figures and Tables

**Table I.** Top 20 components in the alkaloid-NSCLC target network.

Compound	Degree	Stress	Average shortest pathlength	Betweenness centrality	Closeness centrality	(Refs.)
Sevedinine	84	228,576	2.289351852	0.028746974	0.436804853	(77)
Hupehenidine	61	87,532	2.395833333	0.018630794	0.417391304	(86)
(3β,5α,13α,23β)-	57	108,124	2.414351852	0.011970814	0.414189837	(97)
7,8,12,14-tetradehydro-						
5,6,12,13-tetrahydro-						
3,23-dihydroxyveratraman-6-one						
Yubeinine	54	115,480	2.428240741	0.007531494	0.411820782	(37)
Korseveramine	53	114,668	2.43287037	0.008313714	0.411037108	(72)
(20R,25R)-23,26-epimino-	53	47,348	2.43287037	0.014789158	0.411037108	(132)
3β-hydroxy-5α-cholest-						
23(N)-ene-6,22-dione-						
3-0-β-D-glucopyranoside						
Pingbeinone	50	102,036	2.446759259	0.006712711	0.408703879	(64)
Taipaienine	46	94,116	2.465277778	0.00482595	0.405633803	(5)
Sevedinedione	46	78,006	2.465277778	0.008048579	0.405633803	(78)
Ziebeimine	44	80,910	2.474537037	0.004788552	0.404115996	(56)
Pengbeimine C	43	76,382	2.479166667	0.004497064	0.403361345	(104)
Sewertzidine	42	81,864	2.483796296	0.003411146	0.402609506	(73)
Puqienine D	42	69,234	2.483796296	0.004562118	0.402609506	(95)
Pengbeimine A	42	72,362	2.483796296	0.003643213	0.402609506	(101)
Fetisinine	42	44,608	2.483796296	0.009557058	0.402609506	(137)
N-oxide of verticinone	40	54,080	2.493055556	0.009314646	0.401114206	(13)
Hupehenizine	40	70,112	2.493055556	0.003786804	0.401114206	(19)
Ebeienine	40	54,296	2.493055556	0.008436484	0.401114206	(21)
Delafrinone	39	69,290	2.497685185	0.003396726	0.400370714	(49)
Ussurienone	39	49,010	2.497685185	0.005331877	0.400370714	(63)

NSCLC, non-small cell lung cancer.

**Table II.** Cluster analysis of the alkaloid-NSCLC target PPI network.

Cluster	Score	Nodes	Edges	Node targets
1	34.723	48	816	ABL1, AKT1, AR, AURKA, BCL2L1, BRD4, CCNE1, CDK1, CDK2, CDK4, CHEK1, CHUK, EGFR, EP300, ERBB2, ESR1, ESR2, EZH2, FGF2, FLT1, GSK3B, HDAC1, HDAC2, HDAC6, HSP90AA1, IGF1R, IL2, JAK2, JUN, KDR, MAP2K1, MAPK1, MAPK14, MCL1, MDM2, MET, MTOR, PARP1, PGR, PIK3CA, PPARG, PTGS2, PTPRC, RPS6KB1, SRC, STAT3, SYK, XIAP
2	13.053	39	248	ACE, AKT2, ALK, APP, BIRC2, BIRC3, CASP3, CASP7, CDC25A, CREBBP, CYP19A1, ERBB4, FGFR1, FLT, FYN, GRB2, HDAC3, ICAM1, IKBKB, IKBKG, JAK1, JAK3, KIT, LCK, LGALS3, MAPK8, MDM4, NR3C1, NTRK1, PDGFRA, PIK3CB, PIK3CG, PLK1, PRKCA, PRKCD, PRKDC, PTPN1, TP53
3	4.880	26	61	SYK, IKBKB, AURKA, LGALS3, MELK, TTK, PIK3CG, NOS2, CDC45, KIF11, ACE, CHUK, CDC25B, IKBKG, CDK9, CDK7, INSR, NCOR2, CCNE2, YES1, HDAC3, TLR9, AURKB, TYK2, VDR, PIK3CB
4	4.308	14	28	OPRM1, MAOA, CHRNA4, F2R, ACHE, PTGER3, CHRNA3, CHRM2, HTR3A, DNM1, CYP24A1, TBXAS1, DRD2, CYP27B1
5	4.000	4	6	CTSD, CTSL, CTSB, CTSK
6	4.000	4	6	SCD, ACACA, NR1H3, FABP4
7	3.111	10	14	CCR3, CHRNA7, CHRM3, PLA2G2A, CHRM1, PTGER1, PTGFR, LTA4H, PTGER2, PTGES
8	3.000	3	3	PTPN22, IRAK1, TLR7
9	3.000	3	3	CYP3A4, DPP4, NR1H4
10	3.000	3	3	ADRB2, DRD4, CRHR1
11	2.667	7	8	P2RX7. ADORA1, GRIN1, CACNA1G, KCNQ1, KCNK3, GABBR1

NSCLC, non-small cell lung cancer.

## Data Availability

The data generated in the present study may be requested from the corresponding author.
