# Supplementary material for: Mechanism of action of alkaloids in Fritillaria-induced autophagy and apoptosis in non-small cell lung cancer
Source: Oncol Lett. 2026 Apr 15;31(6):243. doi: 10.3892/ol.2026.15598 (PMC13122755; doi:10.3892/ol.2026.15598)
Supplement: Supporting Data [file Supplementary_Data.pdf]

Table SI. Alkaloids identified from *Fritillaria*.

Name	Smiles
Verticine	C[C@H]1CC[C@@]2([H])[C@](O)(C)[C@@]3([H])CC[C@]4([H])[C@@]([H])(C[C@@]5([H])[C@@]4([H])C[C@H2][C@]6([H])[C@]5(C)CC[C@H](O)C6)[C@]3([H])CN2C1
Verticinone	O[C@H]1CC[C@@]2(C)[C@](C(C[C@]3([H])[C@]2([H])C[C@]4([H])[C@@]3([H])CC[C@@]5([H])[C@@]4([H])CN6[C@](CC[C@H](C)C6)([H])[C@]5(O)C)=O)([H])C1
Isoverticine	O[C@H]1CC[C@]2(C)[C@]3([H])[C@@]([C@@](CC[C@@]4([H])[C@@]5([H])CN6[C@](CC[C@H](C)C6)([H])[C@]4(O)C)([H])[C@@]5([H])C3)([H])C[C@@H](O)[C@]([H])2C1
Zhebeinine	O[C@H]1CC[C@]2(C)[C@]3([H])[C@@]([C@@](CC[C@@]4([H])[C@@]5([H])CN6[C@](CC[C@@H](C)C6)([H])[C@]4(O)C)([H])[C@@]5([H])C3)([H])C[C@H](O)[C@]([H])2C1
Taipaienine	C[C@@]1(O)CC[C@]2([H])[C@H](C)[C@]3([H])CC[C@@]4([H])[C@]5([H])CC([C@@]6([H])C[C@@H](O)CC[C@]6(C)[C@@]5([H])C[C@@]4([H])[C@]3([H])CN2C1)=O
Dongbeirine	C[C@@H]1CC[C@@]2(C)[C@@H](C)[C@@]3(C)CC[C@@]4(C)[C@]5(C)CC([C@@]6([H])C[C@@H](O)CC[C@]6(C)[C@@]5(C)C[C@@]4(C)[C@]3(C)CC2C1)=O
Ebeiedinone	C[C@H]1CC[C@H]2[C@@H]([C@H]3CC[C@@H]4[C@H]([C@@H]3CN2C1

	)C[C@H]5[C@H]4CC(=O)[C@@H]6[C@@]5(CC[C@@H](C6)O)C)C
Forticine	C[C@H]1CC[C@@]2(C)[C@@H](C)[C@@]3(C)CC[C@@]4(C)[C@]5(C)C[C@@H](C)[C@@]6(C)C[C@@H](C)CC[C@]6(C)[C@@]5(C)C[C@@]4(C)[C@]3(C)CC2C1
Eduardinine	[H][C@]12C[C@@H](O)CC[C@]1(C)[C@@]([H])(C[C@]3([H])[C@@]4([H])CC[C@@]5([H])[C@@]3([H])CN6[C@](CC[C@H](C)C6)([H])[C@@H]5C)[C@@]4([H])C[C@@H]2O
Puqiedine	C[C@@H]1CC[C@@]2([H])[C@H](C)[C@@]3([H])CC[C@@]4([H])[C@]5([H])C[C@@H](O)[C@@]6([H])C[C@@H](O)CC[C@]6(C)[C@@]5([H])C[C@@]4([H])[C@]3([H])CN2C1
Lichuanine	C[C@@H]1CC[C@@]2([H])[C@@H](C)[C@@]3([H])CC[C@@]4([H])[C@]5([H])C[C@@H](O)[C@@]6([H])C[C@@H](O)CC[C@]6(C)[C@@]5([H])C[C@@]4([H])[C@]3([H])CN2C1
Petilidine	C[C@@H]1CC[C@@]2([H])[C@H](C)[C@@]3([H])CC[C@@]4([H])[C@]5([H])C[C@H](O)[C@@]6([H])C[C@@H](O)CC[C@]6(C)[C@@]5([H])C[C@@]4([H])[C@]3([H])CN2C1
N-oxide of verticinone	C[C@H](C1)CC[C@@]2([H])[C@](O)(C)[C@@]3([H])CC[C@@]4([H])[C@]5([H])CC([C@@]6([H])C[C@@H](O)CC[C@]6(C)[C@@]5([H])C[C@@]4([H])[C@]3([H])CN21=O)=O
Edpetilidine	O[C@H]1CC[C@@]2(C)[C@]([C@H](O)C[C@]3([H])[C@]2([H])C[C@]4([H])C3CC[C@@]5([H])[C@@]4([H])CN6[C@](CC[C@H](C)C6)([H])[C@@H]5C)(

	[H])C1
Imperialine	O[C@H]1CC[C@@]2(C)[C@](C(C[C@]3([H])[C@]2([H])C[C@]4([H])[C@@]3([H])CC[C@]5([H])[C@@]4([H])CN6[C@](CC[C@H](C)C6)([H])[C@]5(O)C)=O)([H])C1
Imperiazine	O[C@H]1CC[C@@]2(C)[C@](C(C[C@]3([H])[C@]2([H])C[C@]4([H])[C@@]3([H])CC[C@]5([H])[C@@]4([H])CN6[C@](CC[C@@H](C)C6)([H])[C@]5(O)C)=O)([H])C1
Petine	O[C@H]1CC[C@@]2(C)[C@]([C@H](O)C[C@]3([H])[C@]2([H])C[C@]4([H])[C@@]3([H])CC[C@]5([H])[C@@]4([H])CN6[C@](CC[C@@H](C)C6)([H])[C@]5(O)C)([H])C1
Hupehenine(delavine)	O[C@H]1CC[C@@]2(C)[C@]([C@H](O)C[C@]3([H])[C@]2([H])C[C@]4([H])[C@@]3([H])CC[C@]5([H])[C@@]4([H])CN6[C@](CC[C@H](C)C6)([H])[C@@H]5C)([H])C1
Hupehenizine	O=C1CC[C@@]2(C)[C@]([C@H](O)C[C@]3([H])[C@]2([H])C[C@]4([H])[C@@]3([H])CC[C@]5([H])[C@@]4([H])CN6[C@](CC[C@H](C)C6)([H])[C@@H]5C)([H])C1
Zhebeirine	[H][C@]12[C@]3([H])CC([C@@]4([H])C[C@@H](O)CC[C@]4(C)[C@@]3([H])C[C@@]1([H])[C@@]5([H])[C@]([C@](C)([H])[C@@](CCC(CC)C6)([H])N6C5)([H])CC2)=O
Ebeienine	[H][C@]12[C@]3([H])CC([C@@]4([H])C[C@@H](OC(C)=O)CC[C@]4(C)[C@@]3([H])C[C@@]1([H])[C@@]5([H])[C@]([C@](C)(O)[C@@](CCC(CC)C6)([

	H])N6C5)([H])CC2)=O
Harermine(persicanidine B)	O[C@H]1CC[C@@]2(C)[C@]([C@H](O)C[C@]3([H])[C@]2([H])C[C@]4([H])[C@@]3([H])CC[C@]5([H])[C@@]4([H])CN6[C@](CC[C@@H](C)C6)([H])[C@@H]5C)([H])C1
Siechuansine	O[C@H]1CC[C@@]2(C)[C@]([C@@H](O)C[C@]3([H])[C@]2([H])C[C@]4([H])[C@@]3([H])CC[C@@]5([H])[C@]4([H])CN6[C@@](CC[C@H](C)C6)([H])[C@]5(O)C)([H])C1
(3β,5α,6α)-6,20-dihydroxycevan-3-yl acetate	[H][C@]12[C@]3([H])CC([C@@]4([H])C[C@@H](O)CC[C@]4(C)[C@@]3([H])C[C@@]1([H])[C@@]5([H])[C@]([C@](C)(O)[C@@](CCC(CC)C6)([H])N6C5)([H])CC2)=O
Tortifoline(songbeinine)	O[C@H]1CC[C@@]2(C)[C@]([C@H](O)C[C@]3([H])[C@]2([H])C[C@]4([H])[C@@]3([H])CC[C@]5([H])[C@@]4([H])CN6[C@@](CC[C@H](C)C6)([H])[C@H]5C)([H])C1
Songbeinone	O[C@H]1CC[C@@]2(C)[C@](C(C[C@]3([H])[C@]2([H])C[C@]4([H])[C@@]3([H])CC[C@]5([H])[C@@]4([H])CN6[C@@](CC[C@H](C)C6)([H])[C@H]5C)=O)([H])C1
Hupehenizioside(delavinone-3-O-β-D-glucopyranoside)	[H][C@]12C[C@@H](O[C@]3([H])O[C@H](CO)[C@@H](O)[C@H](O)[C@H]3O)CC[C@]1(C)[C@]4([H])[C@@]([C@@](CC[C@]5([H])[C@@]6([H])CN7[C@@](CC[C@H](C)C7)([H])[C@@H]5C)([H])[C@@]6([H])C4)([H])CC2=O
Hupeheninoside	[H][C@]12C[C@H](O[C@]3([H])O[C@H](CO)[C@@H](O)[C@H](O)[C@H]3O)CC[C@]1(C)[C@]4([H])[C@@]([C@@](CC[C@@]5([H])[C@@]6([H])CN7[

	C@](CC[C@H](C)C7)([H])[C@@H]5C)([H])[C@@]6([H])C4)([H])C[C@@H]2O
Hupehemonoside	[H][C@]12C[C@@H](O[C@]3([H])O[C@H](CO)[C@@H](O)[C@H](O)[C@H]3O)CC[C@]1(C)[C@]4([H])[C@@]([C@@](CC[C@]5([H])[C@@]6([H])CN7[C@@](CC[C@H](C)C7)([H])[C@]5(O)C)([H])[C@@]6([H])C4)([H])CC2=O
Peiminoside	[H][C@]12C[C@@H](O[C@]3([H])O[C@H](CO)[C@@H](O)[C@H](O)[C@H]3O)CC[C@]1(C)[C@]4([H])[C@@]([C@@](CC[C@@]5([H])[C@@]6([H])CN7[C@](CC[C@H](C)C7)([H])[C@]5(O)C)([H])[C@@]6([H])C4)([H])C[C@@H]2O
Yibeinoside A	[H][C@]12C[C@@H](O[C@H]3O[C@@H](CO)[C@H](O)[C@@H](O)[C@@H]3O)CC[C@]1(C)[C@]4([H])[C@@]([C@@](CC[C@]5([H])[C@@]6([H])CN7[C@](CC[C@H](C)C7)([H])[C@@H]5C)([H])[C@@]6([H])C4)([H])CC2=O
Yibeinoside B	[H][C@]12C[C@@H](O[C@H]3[C@H](O)[C@@H](O)[C@H](O)[C@@H](CO)O3)CC[C@]1(C)[C@]4([H])[C@@]([C@@](CC[C@]5([H])[C@@]6([H])CN7[C@](CC[C@H](C)C7)([H])[C@@H]5C)([H])[C@@]6([H])C4)([H])CC2=O
Delavinone	C[C@H]1CC[C@H]2[C@@H]([C@@H]3CC[C@@H]4[C@H]([C@@H]3CN2C1)C[C@H]5[C@H]4CC(=O)[C@@H]6[C@@]5(CC[C@@H](C6)O)C)C
Baimonidine	O[C@@H]1CC[C@@]2(C)[C@]([C@H](O)CC3[C@]2([H])C[C@]4([H])[C@@]3([H])CC[C@@]5([H])[C@@]4([H])CN6[C@](CC[C@H](C)C6)([H])[C@]5(O)C)([H])C1
Isobaimonidine	O[C@H]1CC[C@@]2(C)[C@]([C@@H](O)C[C@]3(C)[C@]2([H])C[C@]4([H])

	[C@@]3([H])CC[C@]5([H])[C@@]4([H])CN6[C@](CC[C@H](C)C6)([H])[C@]5(O)C)([H])C1
Hupeheninate	[H][C@]12C[C@@H](OC(C)=O)CC[C@]1(C)[C@]3([H])[C@@]([C@@](CC[C@]4([H])[C@@]5([H])CN6[C@](CC[C@H](C)C6)([H])[C@@H]4C)([H])[C@@]5([H])C3)(C)C[C@H]2O
Hupehenirine	O[C@H]1CC[C@@]2(C)[C@](C(C[C@]3(C)[C@]2([H])C[C@]4([H])[C@@]3([H])CC[C@]5([H])[C@@]4([H])CN6[C@](CC[C@H](C)C6)([H])[C@@H]5C)=O)([H])C1
Yubeinine	O[C@@H]1CC[C@@]2(C)[C@](C(C[C@]3(C)[C@]2([H])C[C@]4([H])[C@@]3([H])CC[C@]5([H])[C@@]4([H])CN6[C@](CC[C@H](C)C6)([H])[C@]5(O)C)=O)([H])C1
Puqiedinone-3-O-b-D-glucopyranoside	[H][C@@]12[C@]([C@@H](C)[C@@](CC[C@@H](C)C3)([H])N3C2)([H])CC[C@@]4([H])[C@]5([H])CC([C@@]6([H])C[C@@H](O[C@]7([H])O[C@H](CO)[C@@H](O)[C@H](O)[C@H]7O)CC[C@]6(C)[C@@]5([H])C[C@]41[H])=O
Yubeiside	O=C1CC[C@@]2(C)[C@]([C@H](O[C@@]3([H])[C@H](O)[C@@H](O)[C@H](O)[C@@H](CO)O3)C[C@]4(C)[C@]2([H])C[C@]5([H])[C@@]4([H])CC[C@@]6([H])[C@@]5([H])CN7[C@](CC[C@H](C)C7)([H])[C@@H]6C)([H])C1
Petilinine	O[C@@H]1CC[C@@]2(C)[C@]([C@@H](O)C[C@]3(C)[C@]2([H])C[C@]4([H])[C@@]3([H])CC[C@@]5([H])[C@@]4([H])CN6[C@](CC[C@@H](C)C6)([H])[C@@H]5C)([H])C1
Verticine N-oxide	O[C@H]1CC[C@@]2(C)[C@]([C@@H](O)C[C@]3([H])[C@]2([H])C[C@]4([

	H])[C@@]3([H])C[C@@H](C)C5[C@@]4([H])C[N+]6([O-])[C@](CC[C@H](C)C6)([H])[C@]5(O)C)([H])C1
Imperialine N-oxide	O[C@H]1CC[C@@]2(C)[C@](C(C[C@]3([H])[C@]2([H])C[C@]4([H])[C@@]3([H])CC([H])C5[C@@]4([H])C[N+]6([O-])[C@](CC[C@H](C)C6)([H])[C@]5(O)C)=O)([H])C1
Petine N-oxide	O[C@H]1CC[C@@]2(C)[C@]([C@H](O)C[C@]3([H])[C@]2([H])C[C@]4([H])[C@@]3([H])CC([H])C5[C@@]4([H])C[N+]6([O-])[C@](CC[C@@H](C)C6)([H])[C@]5(O)C)([H])C1
Isoverticine-β-N-oxide	O[C@H]1CC[C@@]2(C)[C@]([C@H](O)C[C@]3([H])[C@]2([H])C[C@]4([H])[C@@]3([H])CC([H])C5[C@@]4([H])C[N+]6([O-])[C@](CC[C@H](C)C6)([H])[C@]5(O)C)([H])C1
Lichuanisinine	O[C@H]1CC[C@@]2(C)[C@]([C@H](O)C[C@]3([H])[C@]2([H])C[C@]4([H])[C@@]3([H])CC([H])C5[C@@]4([H])C[N+]6([O-])[C@](CC[C@H](C)C6)([H])[C@H]5C)([H])C1
Zhebeininoside	C[C@H]1O[C@](O[C@@H]2C[C@@]([C@@H](O)C[C@]3([H])[C@]4([H])CC5[C@@]3([H])CC[C@@]6([H])[C@@]5([H])CN7[C@@](CC[C@@H](C)C7)(C)[C@@]6(C)O)([H])[C@]4(C)CC2)([H])[C@H](O)[C@@H](O)[C@@H]1O.CO
Delafrine	C[C@@]12[C@@](C(O)C[C@]3([H])[C@]2([H])C[C@]4([H])[C@@]3([H])C[C@H](O)[C@@]5([H])[C@@]4([H])CN6[C@](CC[C@H](C)C6)([H])[C@@H]5C)([H])C[C@@H](O)CC1

Ddelafrinone	O[C@H]1CC[C@@]2(C)[C@](C(C[C@]3([H])[C@]2([H])C[C@]4([H])[C@@]3([H])C[C@H](O)[C@@]5([H])[C@@]4([H])CN6[C@](CC[C@H](C)C6)([H])[C@@H]5C)=O)([H])C1
Pingbeimine B	O[C@H]1CC[C@@]2(C)[C@]([C@@H](O)C[C@]3([H])[C@]2([H])C[C@]4(O)[C@@]3(O)C[C@H](O)[C@]5([H])[C@@]4([H])CN6[C@](CC[C@H](C)C6)([H])[C@]5(O)C)([H])C1
Pingbeimine C	O[C@H]1CC[C@@]2(C)[C@](C([C@@H](O)[C@]3([H])[C@]2([H])C[C@]4([H])[C@@]3(O)C[C@H](O)[C@@]5([H])[C@@]4([H])CN6[C@](CC[C@H](C)C6)([H])[C@]5(O)C)=O)([H])C1
Persicanidine A	O[C@H]1CC[C@@]2(C)[C@]([C@H](O)C[C@]3([H])[C@]2([H])C[C@]4([H])[C@@]3([H])CC[C@]5([H])[C@]4([H])CN6[C@](CC[C@@H](C)C6)([H])[C@@H]5C)([H])C1
Fritillarizine(solanidine)	O[C@@H](C1)CC[C@@]2(C)C1=CC[C@]3([H])[C@]2([H])C[C@]4([H])[C@@]3([H])CC[C@@]5([H])[C@@]4([H])CN6[C@](CC[C@H](C)C6)([H])[C@]5(O)C
Shinonomenine	O[C@H](C1)CC[C@@]2(C)C1=CC[C@]3([H])[C@]2([H])C[C@]4([H])[C@@]3([H])CC[C@@]5([H])[C@@]4([H])CN6[C@](CC[C@H](C)C6)([H])[C@H]5C
Iso-shinonomenine	O[C@H](C1)CC[C@@]2(C)C1=CC[C@]3([H])[C@]2([H])C[C@]4([H])[C@@]3([H])CC[C@@]5([H])[C@@]4([H])CN6[C@](CC[C@H](C)C6)([H])[C@@H]5C
Ziebeimine	O[C@@H]1CC[C@@]2(C)[C@]([C@H](O)C[C@]3([H])[C@]2([H])C[C@]4([

	H])[C@@]3([H])CCC5=C4CN6[C@](CC[C@@H](C)C6)([H])[C@@H]5C)([H])C1
Ebeinone	O[C@H]1CC[C@@]2(C)[C@](C(C[C@]3([H])[C@]2([H])C[C@]4([H])[C@@]3([H])CCC5=C4CN6[C@](CC[C@@H](C)C6)([H])[C@@H]5C)=O)([H])C1
Impericine	O[C@H]1CC[C@]2(C)[C@]3([H])[C@@]([C@@](C[C@H](O)[C@@]4([H])[C@@]5([H])CN6[C@](C=C[C@H](C)C6)([H])[C@@H]4C)([H])[C@@]5([H])C3)([H])C[C@@H](O)[C@]([H])2C1
Heilonine	O[C@H]1CC[C@]2(C)[C@]3([H])[C@@](C4=CC=C([C@@H](C)[C@@](CC[C@@H](C)C5)([H])N5C6)C6=C4C3)([H])C[C@@H](O)[C@]([H])2C1
Ussuriedine	O[C@H]1CC[C@]2(C)[C@]3([H])[C@@](C4=CC=C([C@H](C)C(CCC5C6)N6[C@]7(C5)O)C7=C4C3)([H])C[C@@H](O)[C@@]2([H])C1
Ussuriedinone	O[C@H](C[C@]12[H])CC[C@]2(C)[C@]3([H])[C@@](C4=CC=C([C@H](C)C(CCC5C6)N6[C@]7(C5)O)C7=C4C3)([H])CC1=O
Ussurienine	O[C@H]1CC[C@]2(C)[C@]3([H])[C@@](C(C=CC4=C5[C@](OC)(C6)[N@@]7[C@](CC[C@@]6([H])C7)([H])[C@@H]4C)=C5C3)([H])C[C@@H](O)[C@@]2([H])C1
Ussurienone	O[C@H](C[C@]12[H])CC[C@]2(C)[C@]3([H])[C@@](C(C=CC4=C5[C@](OC)(C6)[N@@]7[C@](CC[C@H]6C7)([H])[C@@H]4C)=C5C3)([H])CC1=O
Pingbeinone	C[C@H]1CC[C@]([C@H](C)[C@@]2([H])[C@]3([H])[C@@](C4)(O)[C@@]([C@@](C5)([H])[C@@]4([H])[C@]6(C)[C@@](C[C@@H](O)CC6)([H])C5=O)([H])CC2)([H])N3C1

Korseverine	O[C@H](C[C@]12[H])CC[C@]2(C)C3=C([C@@](CC[C@@]4([H])[C@@]5([H])CN6[C@](CC[C@H](C)C6)([H])[C@@H]4C)([H])[C@@]5([H])C3)CC1=O
Korsine	O[C@H]1CC[C@]2(C)C3=C([C@@](CC[C@@]4([H])[C@@]5([H])CN6[C@]([C@H](O)C[C@@H](C)C6)([H])[C@@H]4C)([H])[C@@]5([H])C3)C[C@@H](O)[C@]([H])2C1
Korseveriline	O[C@@H]1CC[C@]2(C)[C@]3([H])[C@@]([C@@](CC[C@@]4([H])[C@@]5([H])CN6[C@](CC[C@@H](C)C6)([H])[C@@H]4C)(O)[C@@]5([H])C3)([H])C[C@@H](O)[C@]([H])2C1
Edpetisine	O=C1CC[C@]2(C)C3=C([C@@](CC[C@@]4([H])[C@@]5([H])CN6[C@](CC[C@H](C)C6)([H])[C@]4(O)C)([H])[C@@]5([H])C3)CC[C@]([H])2C1
Korselidine	O[C@H]1CC[C@]2(C)[C@]3([H])[C@@]([C@@](CC[C@@]4([H])[C@@]5([H])CN6[C@](CC[C@@H](C)C6)([H])[C@]4(O)C)([H])[C@@]5([H])C3)([H])C[C@@H](O)[C@]([H])2C1
Edpetisidine	O[C@@H]1CC[C@]2(C)C3=C([C@@](CC[C@@]4([H])[C@@]5([H])CN6[C@](CC[C@@H](C)C6)([H])[C@]4(O)C)([H])[C@@]5([H])C3)C[C@@H](O)[C@]([H])2C1
Korseveridine	O[C@H]1CC[C@]2(C)[C@@]3([H])C[C@]4([H])C([C@H](O)C[C@@]5([H])[C@@]4([H])CN6[C@](CC[C@@H](C)C6)([H])[C@@H]5C)=C3CC[C@]([H])2C1
Korseveramine	O[C@@H]1CC[C@]2(C)[C@@]3([H])C[C@]4([H])C([C@@H](O)C[C@@]5([H])[C@@]4([H])CN6[C@](CC[C@@H](C)C6)([H])[C@@H]5C)[C@]3([H])C[

	C@H](O)[C@]([H])2C1
Sewertzidine	O[C@H]1CC[C@]2(C)[C@@]3([H])C[C@]4([H])[C@](CC[C@@]5([H])[C@@]4([H])CN6[C@](CC[C@@H](C)C6)([H])[C@@H]5C)(O)[C@]3([H])C[C@@H](O)[C@]([H])2C1
Persicanidine B	C[C@@H]1CC[C@H]2C([C@@H]3CC[C@@H]4[C@H]([C@@H]3CN2C1)C[C@H]5[C@H]4C[C@H]([C@@H]6[C@@]5(CC[C@@H](C6)O)C)O)C
Edpetisinine	O[C@@H]1CC[C@]2(C)[C@]3([H])[C@@]([C@@]([C@@H](O)C[C@@]4([H])[C@@]5([H])CN6[C@](CC[C@H](C)C6)([H])[C@@H]4C)([H])[C@@]5([H])C3)([H])C[C@@H](O)[C@]([H])2C1
Severine N-oxide	C[C@]12CC[C@@H](OC(C)=O)C[C@@]([H])1[C@H](O)C[C@]3([H])[C@]2([H])C[C@]4([H])[C@@]3(O)CC[C@@]5(C)[C@@]4([H])C[N+]6([O-])[C@](CC[C@@H](C)C6)([H])[C@@H]5C
Sevedinine	O=C1CC[C@]2(C)[C@]3([H])[C@@]([C@@](CC[C@@]4(C)[C@@]5([H])CN6[C@](CC[C@@H](C)C6)([H])[C@@H]4C)([H])[C@@]5(O)C3)([H])C[C@@H](O)[C@]([H])2C1
Sevedinedione	O=C(C[C@]12[H])CC[C@]2(C)[C@]3([H])[C@@]([C@@](CC[C@@]4([H])[C@@]5([H])CN6[C@](CC[C@@H](C)C6)([H])[C@@H]4C)(O)[C@@]5([H])C3)([H])CC1=O
Chuanbeinone	C[C@H]1CC[C@]2([H])[C@H](C)[C@]3([H])CC[C@@]4([H])[C@]5([H])CC([C@@]6([H])C[C@@H](O)CC[C@]6(C)[C@@]5([H])C[C@@]4([H])[C@]3([H])CN2C1)=O

Siechuantine	O[C@H]1CC[C@]2(C)C3([H])[C@@](C(CC[C@@]4([H])[C@]([H])5O[C@@]67[C@](NCC(C)[C@@H]6O7)([H])[C@H]4C)(O)C5(C)C3)([H])CCC([H])2C1
Pingpeimine A	O[C@H]1CC[C@]2(C)[C@]3([H])[C@@]([C@@](C[C@H](O)[C@@]4([H])[C@@]5([H])CN6[C@](CC[C@H](C)C6)([H])[C@]4(O)C)(O)[C@@]5([H])C3)([H])C[C@H](O)[C@]([H])2C1
Walujewine E	[H][C@@]12[C@@]([C@@H](C)[C@@](CC[C@H](C)C3)([H])N3C2)([H])CC[C@]4([H])[C@]1(C[C@@]([C@]5(C)[C@]6([H])C[C@@H](O[C@]7([H])O[C@H](CO)[C@@H](O[C@@]8([H])[C@H](O)[C@@H](O)[C@H](O)[C@@H](CO)O8)[C@H](O)[C@H]7O)CC5)([H])[C@@]4([H])CC6=O)[H]
Walujewine D	[H][C@@]12[C@@]([C@H](C)[C@](CC[C@H](C)C3)([H])N3C2)([H])CC[C@]4([H])[C@]1(C[C@@]5([H])[C@@]4([H])C[C@H](O)[C@]6([H])[C@]5(C)CC[C@H](O[C@]7([H])O[C@H](CO)[C@@H](O)[C@H](O)[C@H]7O)C6)[H]
Walujewine C	[H][C@@]12[C@@]([C@H](C)[C@](CC[C@H](C)C3)([H])N3C2)([H])CC[C@]4([H])[C@]1(C[C@@]5([H])[C@@]4([H])C[C@H](O)[C@]6([H])[C@]5(C)CC[C@H](O)C6)[H]
Walujewine B	[H][C@@]12[C@@]([C@H](C)[C@](CC[C@H](C)C3)([H])N3C2)([H])CC[C@]4([H])[C@]1(C[C@@]5([H])[C@@]4([H])C[C@@H](O)[C@]6([H])[C@]5(C)CC[C@H](O[C@]7([H])O[C@H](CO)[C@@H](O)[C@H](O)[C@H]7O)C6)[H]
Hupehenidine	O[C@H](C1)CC[C@]([C@@]1([H])[C@H](O)C2)(C)[C@@H](C)[C@]2([H])[C@]3([H])CC[C@]([C@]4([H])C3)([H])[C@@H](C)[C@@]5([H])N(C[C@@H](C)CC5)C4=O

Ningpeisine	CN1C[C@H](C)CC[C@@]1([H])[C@@H](C)[C@]2([H])CC[C@@]3([H])[C@]4([H])CC([C@@]5([H])C[C@@H](O)CC[C@]5(C)[C@@]4([H])C[C@@]3([H])[C@H]2C)=O
Ningpeisinoside	CN1C[C@H](C)CC[C@@]1([H])[C@@H](C)[C@]2([H])CC[C@@]3([H])[C@]4([H])CC([C@@]5([H])C[C@@H](OC6[C@H]([C@@H]([C@H]([C@H](CO)O6)O)O)O)CC[C@]5(C)[C@@]4([H])C[C@@]3([H])[C@H]2C)=O
Impranine	C[C@H]1CC[C@@]([C@@H](C)[C@]2([H])C=C[C@]([C@@](CC([C@]3([H])[C@]4(C)CC[C@H](O)C3)=O)([H])[C@]4([H])C5)([H])[C@]5(C)C2)([H])N(C)C1
Dihydroimpranine	C[C@H]1CC[C@@]([C@@H](C)[C@]2([H])C=C[C@]([C@@](C[C@@H](O)[C@]3([H])[C@]4([H])CC[C@H](O)C3)([H])[C@]4([H])C5)([H])[C@]5(C)C2)([H])N(C)C1
Suchengbeisine	O=C1C[C@@]2([H])[C@]([C@@]3([H])C[C@]2([H])[C@]4(C)[C@]1([H])C[C@@H](O)CC4)([H])CC[C@@]5([H])[C@@]3(C)OC[C@@H]5[C@@H]6NC[C@@H](C)CC6
Puqienine A	CN1C[C@H](C)C[C@@H](O)[C@@]1([H])[C@H](C)[C@]2([H])CC[C@]([C@@](C[C@@H](O)[C@]3([H])[C@]4(C)CC[C@H](O)C3)([H])[C@]4([H])C5)([H])C5=C2C
Puqienine B	CN1C[C@H](C)C[C@@H](O)[C@@]1([H])[C@H](C)[C@]2([H])CC[C@]([C@@](CC([C@]3([H])[C@]4(C)CC[C@H](O)C3)=O)([H])[C@]4([H])C5)([H])C5=C2C

Puqienine C	CN1C[C@H](C)C[C@@H](O)[C@@]1([H])[C@H](C)[C@]2([H])CC[C@](C([C@]3([H])C4)=CC([C@]5([H])[C@]3(C)CC[C@H](O)C5)=O)([H])[C@]4([H])[C@]2(C)O
Puqienine D	CN1C[C@H](C)C[C@@H](O)[C@@]1([H])[C@H](C)[C@]2([H])CC=C([C@@](CC([C@]3([H])[C@]4(C)CC[C@H](O)C3)=O)([H])[C@]4([H])C5)[C@]5([H])[C@]2(C)O
Puqienine E	CN1C[C@H](C)C[C@@H](O)[C@@]1([H])[C@H](C)[C@@]2([H])CC=C3[C@@](CC([C@]4([H])[C@]5(C)CC[C@H](O)C4)=O)([H])[C@]5([H])C[C@@]3([H])[C@]2(C)O
(3β,5α,13α,23β)-7,8,12,14-tetradehydro-5,6,12,13-tetrahydro-3,23-dihydroxyveratraman-6-one	C[C@H]1C[C@@H](O)[C@@]([C@@H](C)[C@]2([H])CCC(C([C@]3([H])C4)=CC([C@]5([H])[C@]3(C)CC[C@H](O)C5)=O)=C4[C@]2(O)C)([H])NC1
(3β,5α,13α,23β)-7,8,12,14-tetradehydro-5,6,12,13-tetrahydro-3,23-dihydroxyveratraman-6-one	C[C@H](CN1)C[C@@H](O)[C@]1(C)[C@@H](C)[C@]([C@]2(C)[H])([H])CCC3=C2C[C@@]4([H])C3=CC([C@@]5([H])C[C@@H](O)CC[C@@]54C)=O
Puqienine F	CC1(C)[C@@]([C@@H](C)[C@]2([H])[C@H](O)C[C@H](C)CN2C)([H])[C@]3([H])C[C@@]4([H])[C@@]5([H])CC([C@@]6([H])C[C@@H](O)CC[C@]6(C)[C@@]5([H])C[C@@]41O3)=O
Korsevine	C[C@H]1CC[C@@]([C@@H](C)[C@]2([H])CC[C@]([C@@](CC([C@]3([H])[C@]4(C)CC[C@H](O)C3)=O)([H])[C@]4([H])C5)([H])C5=C2C)([H])N(C)C1
Pengbeimine A	O=C1[C@]2([H])[C@@](CC[C@H](O)C2)(C)[C@]3([H])[C@@]([C@@](CC[C

	@@]4([H])[C@@]5(C)O[C@@]6(CC[C@H](C)CN6)[C@@H]4C)([H])[C@]5([H])C3)([H])C1
Pengbeimine B	C[C@H](CN1C)CC[C@@]21[C@H](C)[C@@]3([H])CC[C@@]4([H])[C@]5([H])CC([C@@]6([H])C[C@@H](O)CC[C@]6(C)[C@@]5([H])C[C@]4([H])[C@]3(C)O2)=O
Pengbeimine D	C[C@H](CN1C)CC[C@@]21[C@@H](C)[C@@]3([H])CC[C@@]4([H])[C@]5([H])CC([C@@]6([H])C[C@@H](O)CC[C@]6(C)[C@@]5([H])C[C@]4([H])[C@]3(C)O2)=O
Pengbeimine C	O=C1C[C@@]([C@](CC2)([H])C3=C(C)[C@@H]2[C@H](C)[C@H]4[C@H](C[C@@H](C)CN4C)O)([H])[C@](C3)([H])[C@@]5(C)[C@@]1([H])C[C@H](O)CC5
Peimisine	[H][C@]1([C@H]2C)[C@](C[C@H](C)CN1)([H])O[C@@]32CC[C@@]4([H])[C@]5([H])CC([C@@]6([H])C[C@@H](O)CC[C@]6(C)[C@@]5([H])CC4=C3C)=O
Yibeissine	[H][C@]1([C@H]2C)[C@](C[C@H](C)CN1)([H])O[C@@]32CC[C@@]4([H])[C@]5([H])CC([C@@]6([H])C[C@@H](O)CC[C@]6(C)[C@@]5([H])[C@@H](O)C4=C3C)=O
Tortifolisine	[H][C@]1([C@H]2C)[C@](C[C@@H](C)CN1)([H])O[C@@]32CC[C@@]4([H])[C@]5([H])CC([C@@]6([H])C[C@@H](O)CC[C@]6(C)[C@@]5([H])CC4=C3C)=O
Hupehenisine	[H][C@]1([C@H]2C)[C@](C[C@H](C)CN1)([H])O[C@@]32CC[C@@](C4=C3

	C)([H])[C@]5([H])CC[C@@]6([H])C[C@@H](O)CC[C@]6(C)[C@@]5([H])C4=O
Songbeisine	[H][C@]1([C@@H]2C)[C@](C[C@H](C)CN1)([H])O[C@@]32CC[C@@](C4=C3C)([H])[C@]5([H])CC[C@@]6([H])C[C@@H](O)CC[C@]6(C)[C@@]5([H])C4=O
23-isokuroyunrinidine	[H][C@]1([C@H]2C)[C@@](C[C@H](C)CN1)([H])O[C@@]32CC[C@@]4([H])[C@]5([H])C[C@@H](O)[C@@]6([H])C[C@H](O)[C@@H](O)C[C@]6(C)[C@@]5([H])CC4=C3C
Kuroyunrinidine	[H][C@]1([C@@H]2C)[C@](C[C@H](C)CN1)([H])O[C@@]32CC[C@@]4([H])[C@]5([H])C[C@@H](O)[C@@]6([H])C[C@H](O)[C@@H](O)C[C@]6(C)[C@@]5([H])CC4=C3C
Peimisine-3-O-b-D-glucopyranoside	C[C@@]12[C@](C(C[C@]3([H])[C@]2([H])CC4=C(C)[C@@]5(O[C@@](C[C@H](C)NC6)([H])[C@]6(C)[C@H]5C)CC[C@@]34[H])=O)([H])C[C@@H](O[C@]7([H])O[C@H](CO)[C@@H](O)[C@H](O)[C@H]7O)CC1
Walujewine A	[H][C@]1([C@H]2C)[C@](C[C@H](C)CN1)([H])O[C@@]32CC[C@@]4([H])[C@]5([H])CC([C@@]6([H])C[C@@H](O)CC[C@]6(C)[C@@]5([H])C[C@@]4(O)C3=C)=O
O-β-D-glucopyranosyl-(1→)-β-D-xylopyranoside	O[C@H]([C@H]1O)[C@H](O[C@]2([H])O[C@H](CO)[C@@H](O)[C@H](O)[C@H]2O)CO[C@@]1([H])O[C@@H]3CC([C@]4(C)CC3)=CC[C@@]([C@]4([H])CC5)([H])C([C@@]5(C)[C@@]6([H])[C@H](C)[C@@]7([H])CC[C@H](C)CN7C6)=C

(22R,25s)-solanid-5-enine-3β,5α,6β-triol 15,16-seco-22αH,25βH-solanida-5,14-dien-3β-ol	O[C@H]1CC[C@@]2(C)[C@]([C@H](O)C[C@]3([H])[C@]2([H])CC[C@@]4(C)[C@@]3([H])C[C@@](N5[C@@]([H])6CC[C@H](C)C5)([H])[C@]4([H])[C@@H]6C)(O)C1
Camtschatcanidine	O[C@H](C1)CC[C@@]2(C)C1=CC[C@]3([H])[C@]2([H])CC[C@@]4(C)[C@@]3([H])C[C@@](N5[C@@]([H])6CC[C@H](CO)C5)([H])[C@]4([H])[C@@H]6C
Solanidine	O[C@H](C1)CC[C@@]2(C)C1=CC[C@]3([H])[C@]2([H])CC[C@@]4(C)[C@@]3([H])C[C@@](N5[C@@]([H])6CC[C@H](C)C5)([H])[C@]4([H])[C@@H]6C
Solanidine 3-O-α-L-rhamnopyranosyl-(1→2)-[β-D-glucopyranosyl-(1→4)-]β-D-glucopyranoside	C[C@]12C(C[C@@H](O[C@]3([H])O[C@H](CO)[C@@H](O[C@]4([H])O[C@H](CO)[C@@H](O)[C@H](O)[C@H]4O)[C@H](O)[C@H]3O[C@@]5([H])[C@H](O)[C@H](O)[C@@H](O)[C@H](C)O5)CC2)=CC[C@]6([H])[C@]1([H])CC[C@@]7(C)[C@@]6([H])C[C@@](N8[C@@]([H])9CC[C@H](C)C8)([H])[C@]7([H])[C@@H]9C
(22S,25S)-Solanid-5-en-3β-ol	C[C@@H]1CN2[C@@]3([H])C[C@@]4([H])[C@]5([H])CC=C6C[C@@H](O)CC[C@]6(C)[C@@]5([H])CC[C@]4(C)[C@@]3([H])[C@H](C)[C@]2([H])CC1
(22S,25S)-Solanid-5,20(21)-dien-3β-ol	C[C@@H]1CN([C@@]2([H])CC1)[C@@]3([H])C[C@@]4([H])[C@]5([H])CC=C6C[C@@H](O)CC[C@]6(C)[C@@]5([H])CC[C@]4(C)[C@@]3([H])C2=C
Hapepunine	O[C@H]1C[C@@]2([H])[C@]3([H])CC=C4C[C@@H](O)CC[C@]4(C)[C@@]3([H])CC[C@]2(C)[C@@]1([H])[C@H](C)[C@]5([H])CC[C@H](C)CN5C
Anrakorinine	O[C@H]1C[C@@]2([H])[C@]3([H])CC=C4C[C@@H](O)CC[C@]4(C)[C@@]

	3([H])CC[C@]2(CO)[C@@]1([H])[C@H](C)[C@]5([H])CC[C@H](C)CN5C
Pingbeinine	O[C@H]1C[C@@]2([H])[C@]3([H])CC=C4C[C@@H](O)CC[C@]4(C)[C@@]3([H])CC[C@]2(C)[C@@]1([H])[C@H](C)[C@@]5([H])CC[C@@](O)(C)CN5C
Pingbeiinoside	O[C@H]1C[C@@]2([H])[C@]3([H])CC=C4C[C@@H](O[C@]5([H])O[C@H](CO)[C@@H](O)[C@H](O)[C@H]5O)CC[C@]4(C)[C@@]3([H])CC[C@]2(C)[C@@]1([H])[C@H](C)[C@@]6([H])CC[C@@](O)(C)CN6C
Hapepunine 3-0-β-cellobioside	O[C@H]1C[C@@]2([H])[C@]3([H])CC=C4C[C@@H](O[C@]5([H])O[C@H](CO)[C@@H](O[C@]6([H])O[C@H](CO)[C@@H](O)[C@H](O)[C@H]6O)[C@H](O)[C@H]5O)CC[C@]4(C)[C@@]3([H])CC[C@]2(C)[C@@]1([H])[C@H](C)C7([H])CC[C@H](C)CN7C.C
Pingbeidinoside	O[C@@H]1C[C@@]2([H])[C@]3([H])CC=C4C[C@@H](O)CC[C@]4(C)[C@@]3([H])CC[C@]2(C)[C@@]1([H])[C@@](C)(O)[C@@]5([H])CC[C@](O[C@@H]6O[C@H](CO)[C@@H](O)[C@H](O)[C@H]6O)(C)CN5C
Puqietinone	CC(CN1C)CCC1[C@@H](C)[C@@]2([H])CC[C@@]3([H])[C@]4([H])CC([C@@]5([H])C[C@@H](O)CC[C@]5(C)[C@@]4([H])CC[C@@]32C)=O
Yibeinoside C	C[C@H](CN1)CC[C@]1([H])[C@@H](C)[C@@]2([H])CC[C@@]3([H])[C@]4([H])CC([C@@]5([H])C[C@@H](O[C@]6([H])O[C@H](CO)[C@H](O[C@]7([H])O[C@H](CO)[C@@H](O)[C@H](O)[C@H]7O)[C@H](O)[C@H]6O)CC[C@]5(C)[C@@]4([H])CC[C@@]32C)=O
Puqietinonoside	C[C@H]1CC[C@@H](N(C1)C)[C@@H](C)[C@H]2CC[C@@H]3[C@@]2(CC[

	C@H]4[C@H]3CC(=O)[C@@H]5[C@@]4(CC[C@@H](C5)O[C@H]6[C@@H]([C@H]([C@@H]([C@H](O6)CO)O)O)O)C)C
N-demethylpuqietinone	C[C@H]1CC[C@@H](NC1)[C@@H](C)[C@H]2CC[C@@H]3[C@@]2(CC[C@H]4[C@H]3CC(=O)[C@@H]5[C@@]4(CC[C@@H](C5)O)C)C
（20R,25R）-23，26-epimino-3β-hydroxy-5α-cholest-23(N）-ene-6,22-dione	C[C@@]12[C@@]([H])(CCC2[C@@H](C(C3=NCC(C)C3)=O)C)[C@]4([H])CC([C@@]5([H])C[C@@H](O)CC[C@]5(C)[C@@]4([H])CC1)=O
（20R,25R）-23，26-epimino-3β-hydroxy-5α-cholest-23(N）-ene-6, 22-dione-3-0-β-D-glucopyranoside cbeietinone	C[C@@]12[C@@]([H])(CCC2[C@@H](C(C3=NCC(C)C3)=O)C)[C@]4([H])CC([C@@]5([H])C[C@@H](O[C@]6([H])O[C@H](CO)[C@@H](O)[C@H](O)[C@H]6O)CC[C@]5(C)[C@@]4([H])CC1)=O
（25R）-23，26-epimino-3β-hydroxy-5α-cholest-23(N）-ene-6,22-dione	C[C@@]12[C@@]([H])(CCC2[C@H](C(C3=NCC(C)C3)=O)C)[C@]4([H])CC([C@@]5([H])C[C@@H](O)CC[C@]5(C)[C@@]4([H])CC1)=O
（25R）-23，26-epimino-3β-hydroxy-5α-cholest-23(N）-ene-6,22-dione 3-O-β-D-glucopyranoside	C[C@@]12[C@@]([H])(CCC2[C@H](C(C3=NCC(C)C3)=O)C)[C@]4([H])CC([C@@]5([H])C[C@@H](O[C@]6([H])O[C@H](CO)[C@@H](O)[C@H](O)[C@H]6O)CC[C@]5(C)[C@@]4([H])CC1)=O
25-Isosolafloridine	C[C@H]1CCC([C@@H](C)[C@@]2([H])[C@H](O)C[C@@]3([H])[C@]4([H])CC[C@@]5([H])C[C@@H](O)CC[C@]5(C)[C@@]4([H])CC[C@@]32C)=NC1
Ebeietinone	C[C@H]1CCC([C@@H](C)[C@@]2([H])CC[C@@]3([H])[C@]4([H])CC([C@]

	5(O)C[C@@H](O)CC[C@]5(C)[C@@]4([H])CC[C@@]32C)=O)=NC1
Fetisinine	CC(C=N1)=CC(O)=C1[C@@H](C)[C@@]2([H])CC[C@@]3([H])[C@]4([H])C C([C@@]5([H])C[C@H](O)CC[C@]5(C)[C@@]4([H])CC[C@@]32C)=O
Puqietinedione	C[C@H](CN1C)CC[C@]1([H])[C@@H](C)[C@@]2([H])CC[C@@]3([H])[C@] 4([H])CC([C@@]5([H])CC(CC[C@]5(C)[C@@]4([H])CC[C@@]32C)=O)=O

Table SII. Survival rates of A549 cells treated with eight alkaloids for 48 h.

			Concentration, μM			
Alkaloid	1	5	10	15 μM	30 μM	60 μM
Imperialine	-	-	-	91.88±10.26	90.20±7.6u1	100.27±5.67
Verticinone	93.93±1.86	81.07±7.18[a]	78.03±9.02[b]	78.30±8.71[b]	65.00±11.15[c]	66.77±9.80[c]
Verticine	-	-	-	91.63±11.43	85.73±3.93	101.13±7.43
Imperialine-3-β-D-glucoside	-	-	-	89.78±2.82	90.03±7.44	97.27±5.52
Delavine	101.60±4.35	89.73±2.16	86.50±5.43	83.53±4.33[a]	81.27±6.18[a]	82.43±7.20[a]
Peimisine	-	-	-	97.33±11.21	96.80±11.06	109.40±3.72
Ebeiedinone	94.83±5.85	90.23±1.747	81.87±9.87[a]	71.45±6.69[b]	67.00±5.56[b]	57.73±13.59[b]
Delavinone	-	-	-	93.65±5.28	91.80±7.14	99.47±7.18

[a]$P<0.05$, [b]$P<0.01$, [c]$P<0.001$, vs. control.

Table SIII. Survival rates of NCI-H1299 cells treated with eight alkaloids for 48h (%)

	Concentration, μM					
Alkaloid	1	5	10 μM	15 μM	30 μM	60 μM
Imperialine	-	-	-	90.50±3.25	89.03±5.58	86.35±3.89
Verticinone	95.73±4.21	85.93±5.25	80.40±9.27[a]	75.20±3.80[a]	77.37±9.59[a]	74.90±10.89[b]
Verticine	-	-	-	94.20±5.52	97.20±8.58	93.25±13.93
Imperialine-3-β-D-glucoside	-	-	-	90.20±4.10	99.83±7.81	98.15±10.96
Delavine	97.77±3.41	94.97±3.40	89.10±2.63	85.23±7.46	86.50±4.78	83.05±6.86[a]
Peimisine	-	-	-	95.70±3.68	103.27±6.59	103.35±12.80
Ebeiedinone	95.17±2.64	91.27±8.02	83.80±2.16[a]	82.73±9.34[a]	74.03±3.63[b]	65.10±0.99[c]
Delavinone	-	-	-	99.75±0.636	103.80±5.78	101.15±1.91

[a]P<0.05, [b]P<0.01, [c]P<0.001, vs. control.

Table SIV. Survival rate (%) of NCI-H1975 cells treated with eight alkaloids for 48 h.

	Concentration, μM	
Compound	15	30 μM
Imperialine	116.35±11.24	111.90±2.69
Verticinone	97.50±1.41	95.80±6.65
Verticine	117.05±0.64	114.05±0.49
Imperialine-3-β-D-glucoside	114.80±4.10	114.20±1.84
Delavine	107.15±3.89	101.20±5.94
Peimisine	108.35±3.18	109.80±2.40
Ebeiedinone	92.40±0.71	89.65±8.98
Delavinone	109.25±4.74	106.65±5.02
